# Transcriptional silencing of long noncoding RNA *GNG12-AS1* uncouples its transcriptional and product-related functions

**DOI:** 10.1038/ncomms10406

**Published:** 2016-02-02

**Authors:** Lovorka Stojic, Malwina Niemczyk, Arturo Orjalo, Yoko Ito, Anna Elisabeth Maria Ruijter, Santiago Uribe-Lewis, Nimesh Joseph, Stephen Weston, Suraj Menon, Duncan T. Odom, John Rinn, Fanni Gergely, Adele Murrell

**Affiliations:** 1 Cancer Research UK Cambridge Institute, University of Cambridge, Li Ka Shing Centre, Robinson Way, Cambridge CB2 0RE, UK; 2 Biosearch Technologies Inc., 2199S. McDowell Boulevard, Petaluma, California 94954, USA; 3 Centre for Regenerative Medicine, Department of Biology and Biochemistry, University of Bath, Claverton Down, Bath BA2 7AY, UK; 4 Department of Stem Cell and Regenerative Biology, Harvard University, Cambridge, Massachusetts 02138, USA

## Abstract

Long noncoding RNAs (lncRNAs) regulate gene expression via their RNA product or through transcriptional interference, yet a strategy to differentiate these two processes is lacking. To address this, we used multiple small interfering RNAs (siRNAs) to silence *GNG12-AS1*, a nuclear lncRNA transcribed in an antisense orientation to the tumour-suppressor *DIRAS3*. Here we show that while most siRNAs silence *GNG12-AS1* post-transcriptionally, siRNA complementary to exon 1 of *GNG12-AS1* suppresses its transcription by recruiting Argonaute 2 and inhibiting RNA polymerase II binding. Transcriptional, but not post-transcriptional, silencing of *GNG12-AS1* causes concomitant upregulation of *DIRAS3*, indicating a function in transcriptional interference. This change in *DIRAS3* expression is sufficient to impair cell cycle progression. In addition, the reduction in *GNG12-AS1* transcripts alters MET signalling and cell migration, but these are independent of *DIRAS3*. Thus, differential siRNA targeting of a lncRNA allows dissection of the functions related to the process and products of its transcription.

## Abstract (web summary)

LncRNAs regulate gene expression via their RNA product and through transcriptional interference. Here, Stojic *et al.* uncouple these functions by using multiple siRNAs against GNG12-AS1 to show that this lncRNA has a product related role in MET signaling while its transcription modulates DIRAS3 expression.

## Graphical abstract

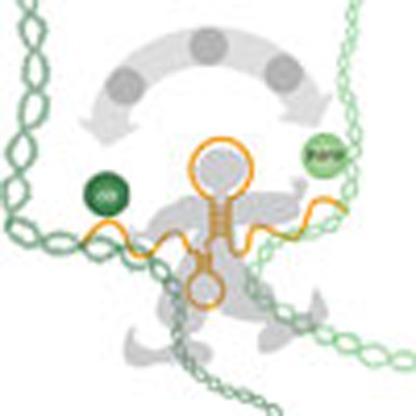

The mammalian genome encodes many long noncoding RNAs (lncRNAs) that play pivotal roles in gene regulation during development and disease pathogenesis^1^,^2^,^3^. LncRNAs can regulate gene expression through transcriptional interference (TI)^4^,^5^ or through the RNA product itself^1^,^2^. TI occurs when the act of transcribing a gene directly interferes with the transcription of an adjacent gene in *cis*, either at its initiation site or at an essential *cis*-regulatory element^6^,^7^. Transcription of lncRNA has been shown to interfere with the transcription of adjacent genes by altering the binding of transcription factors^8^,^9^,^10^ and/or RNA polymerase II (Pol II)^11^, nucleosome remodelling^12^ or inducing changes in histone modifications^13^,^14^.

So far, the strategies for demonstrating TI in mammalian cells have relied upon genetic engineering methodologies that remove the promoter region or insert premature transcriptional termination sites within the transcriptional unit^4^. An alternative approach would be to leave the DNA template intact and directly target the lncRNA with small interfering RNAs (siRNAs). It is widely perceived that RNA interference (RNAi) is a cytoplasmic pathway for post-transcriptional gene silencing^15^. However, RNAi-based technology has been used to post-transcriptionally deplete a lncRNA *Kcnq1ot1* in the nucleus and demonstrate that this lncRNA product is not required to maintain imprinting of adjacent genes^16^. RNAi has also been shown to induce transcriptional gene silencing (TGS)^17^,^18^,^19^. siRNA-directed TGS can lead to epigenetic changes such as DNA methylation and histone methylation at the target promoters^17^,^20^,^21^,^22^. In addition, promoter-targeting siRNAs can induce TGS by blocking the recruitment and the activity of Pol II (refs 23, 24, 25). In all these cases, Argonaute proteins (AGO1 and AGO2) were shown to be key players of TGS^21^,^24^,^26^,^27^,^28^,^29^. Finally, it has been shown that siRNAs can be used to knockdown small-nuclear ncRNA 7SK^30^. These observations prompted us to investigate whether siRNAs can be used to inhibit lncRNA transcription and to explore the functional consequences of this process. In particular, we postulated that by targeting different regions of the lncRNA, we could uncouple the act of transcription from the function of the transcript. This would enable us to investigate how lncRNAs regulate adjacent genes in *cis* through TI. As a model, we used the imprinted tumour-suppressor *DIRAS3* locus, where we have recently characterized a novel lncRNA known as *GNG12-AS1* (ref. 31). *GNG12-AS1* is transcribed in an antisense orientation to *DIRAS3* and its neighbouring non-imprinted genes *GNG12* and *WLS* (Fig. 1a). We have shown that this lncRNA is allele specifically silenced in cancer cell lines depending on the imprinted state of *DIRAS3* (ref. 31). *DIRAS3* (also known as *ARHI* and *NOEY2*) is a Ras-related imprinted tumour suppressor involved in the inhibition of growth, motility and invasion via several signalling pathways including RAS/MAPK, STAT3 and PI3K^32^. *DIRAS3* is downregulated in 70% of breast and ovarian cancer^33^,^34^,^35^, and its loss of expression correlates with cancer progression and metastasis^34^,^35^. The mechanism responsible for *DIRAS3* downregulation to date involves different epigenetic mechanisms and loss of heterozygosity^32^. We hypothesized that TI by *GNG12-AS1* could represent an additional layer of regulating *DIRAS3* dosage.

Here, we demonstrate that *GNG12-AS1* can be transcriptionally silenced with siRNAs complementary to a region proximal to its transcriptional start site (TSS). The transcriptional silencing of *GNG12-AS1*, which is mediated by AGO2, leads to upregulation of *DIRAS3* transcription in *cis*, indicating a function in TI. In contrast, targeting *GNG12-AS1* at the 3′ end did not affect its nascent transcription, but reduced the lncRNA through post-transcriptional gene silencing and, importantly, did not affect *DIRAS3* transcription. We further show different phenotypic effects in cell cycle and migration depending on whether we target the 5′ or 3′ end of *GNG12-AS1.* Altogether, our results demonstrate that strategic targeting of siRNA to different regions of an lncRNA can enable the discrimination between functions related to its active transcription and that of the RNA product.

## Results

### *GNG12-AS1* is a stable lncRNA localized in the nucleus

**Figure 1:**
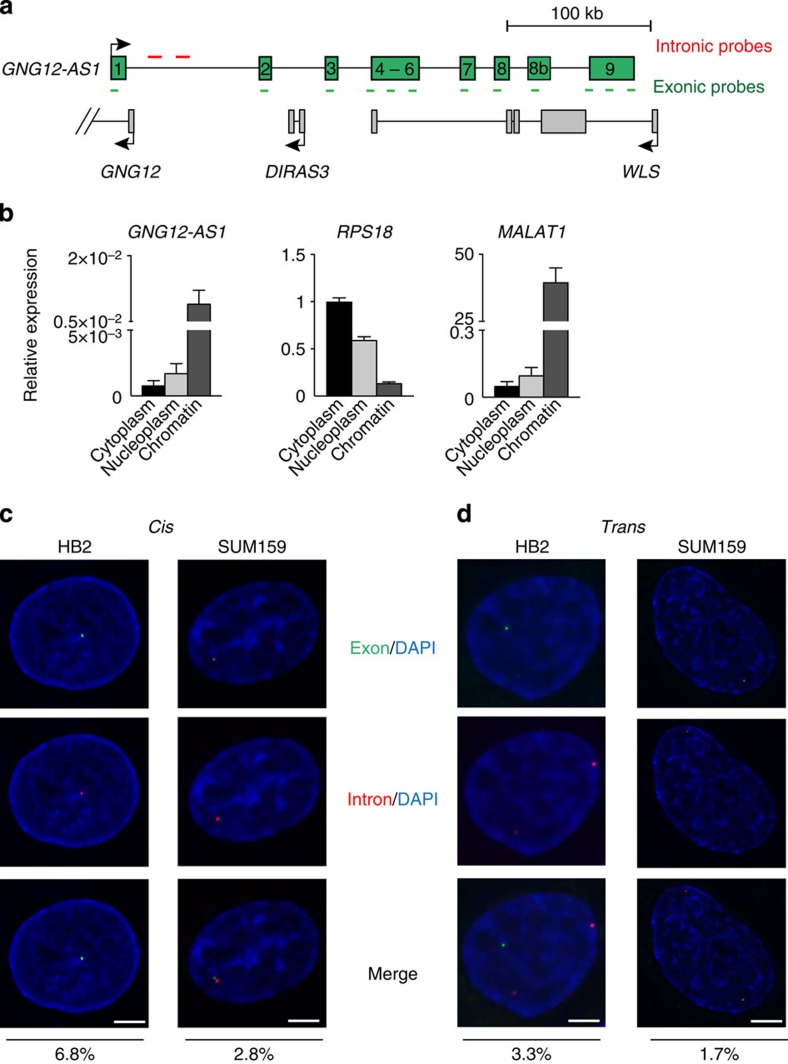
*GNG12-AS1* is a stable lncRNA in the nucleus. (**a**) Schematic representation of the *GNG12-AS1* genomic locus (chr1: 68297971–68668670, hg19) relative to *GNG12, DIRAS3* and *WLS*. The arrows show the direction of transcription. *GNG12-AS1* exons are in green and are numbered as previously reported^31^. Probes used for FISH are marked. Intronic, labelled in red identify the site of transcription. Exonic, labelled in green, mark mature transcripts. (**b**) RNA distribution from the cytoplasm, nucleoplasm and chromatin in SUM159 cells as quantified by qRT–PCR. *RPS18* and *MALAT1* are positive controls for the cytoplasmic and chromatin fraction, respectively. Note enrichment of *GNG12-AS1* in the chromatin fraction. Relative RNA levels are standardized to the geometric mean of *GAPDH* and *β-actin*. Error bars represent the s.e.m. values of three independent experiments. (**c**,**d**) Co-localization of exonic (green) and intronic (red) *GNG12-AS1* probes in HB2 and SUM19 cells by single-molecule RNA FISH. *GNG12-AS1* associates with its site of transcription (2.8% in SUM159, 6.8% in HB2; *cis*, **c**) but is also present at the other sites in the nucleus (1.7% in SUM159, 3.3% in HB2, *trans*, **d**). The numbers represent the percentage of cells positive for exonic and intronic FISH signal (*n*=132 for HB2; *n*=176 for SUM159). The nucleus was stained with 4,6-diamidino-2-phenylindole (DAPI). Scale bar, 7 μm.

In this study, we used three non-cancer cell lines (HB2, HS27, MCF10A), which we have previously shown to have normal imprinted *DIRAS3* expression, and breast cancer cell lines (SUM159, MCF7), where loss of *DIRAS3* imprinting leads to biallelic expression (SUM159) or biallelic silencing of *DIRAS3* (MCF7). The non-cancer cell lines expressed *GNG12-AS1* from both alleles, whereas the cancer cell lines expressed *GNG12-AS1* from one allele^31^. We confirmed the relative expression of *DIRAS3*, *GNG12*, *GNG12-AS1* and *WLS* in these cell lines (Supplementary Fig. 1a). Actinomycin D chase experiments indicate that *GNG12-AS1* is a stable lncRNA with a half-life between 20 and 25 h (Supplementary Fig. 1b). *DIRAS3* expression remained unchanged in HB2 and increased in HS27 when cells were treated with Actinomycin D, suggesting an inverse relationship between *DIRAS3* and *GNG12-AS1* transcription. Despite being a stable lncRNA, *GNG12-AS1* has a low transcript volume (20–80 molecules per 100 cells; Supplementary Fig. 1c). Expression analysis following cell fractionation indicated that *GNG12-AS1* is localized within the chromatin (Fig. 1b), similar to *MALAT1*, a lncRNA known to be associated with chromatin^36^. Using single-molecule RNA fluorescence *in situ* hybridization (RNA FISH; as described in ref. 37), we confirmed that *GNG12-AS1* is nuclear in HB2 and SUM159 cells. Exonic probes complementary to all exons showed that *GNG12-AS1* transcripts accumulate in the nucleus in discrete foci in 19% of SUM159 and 25% of HB2 cells (Supplementary Fig. 1d).

Intronic RNA FISH probes complementary to the first intron of *GNG12-AS1* were found to co-localize with the exonic signals in about two-thirds of cases where both probes were present in the same cell (6.8% in HB2 cells; 2.8% in SUM159 cells; Fig. 1c). As intronic probes are usually indicative of nascent transcription and exonic probes can detect both primary and mature transcripts, their co-localization may indicate that the processed transcript remains at its site of transcription, which would fit with *GNG12-AS1* being co-transcriptionally spliced, as previously reported^31^. However, as we also see separate signals for exonic and intronic probes, it is likely that some *GNG12-AS1* also accumulate at other sites in the nucleus away from its site of transcription (3.3% in HB2 cells; 1.7% in SUM159 cells; Fig. 1d). A caveat to the interpretation of FISH data is that intronic and exonic RNA FISH probes might not hybridize to nascent and mature lncRNA transcripts in a mutually exclusive way. Nascent RNA contains exons and the mature lncRNAs may be present as unspliced isoforms.

In SUM159 cells, the intronic probe mostly produced a single FISH signal (13% of cells being monoallelic, Supplementary Fig. 1e), which fits with previous pyrosequencing data reporting allele-specific *GNG12-AS1* expression in cancer cells^31^. By contrast, in HB2 cells, even though pyrosequencing showed an allelic ratio of 50% (ref. 31), we observed cells with both single and double intronic signals (8 and 6% of cells, respectively; Supplementary Fig. 1e), suggesting that *GNG12-AS1* is heterogeneously biallelic and randomly monoallelic at the cellular level in HB2 cells. Together, these data confirm that *GNG12-AS1* is a stable lncRNA that is present in the nucleus at distinct foci.

### TI between *GNG12-AS1* and *DIRAS3*

**Figure 2:**
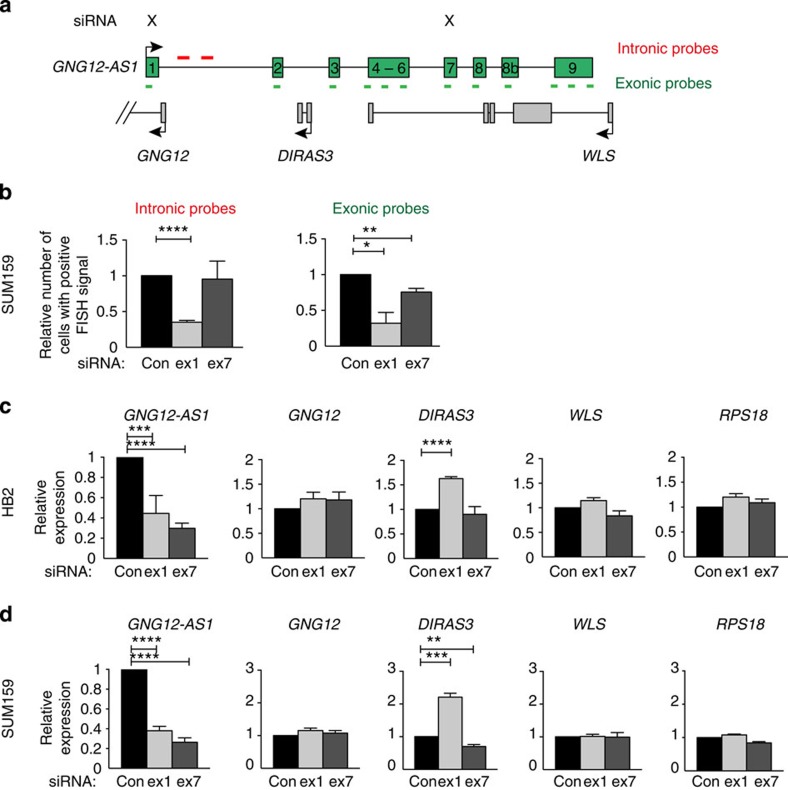
Transcriptional interference by *GNG12-AS1* regulates *DIRAS3*. (**a**) The *GNG12-AS1* locus where X indicates the exons targeted by siRNA. (**b**) Changes in single-molecule RNA FISH signals after treating SUM159 cells with siRNA directed to exon 1 or exon 7 of *GNG12-AS1*. Changes in nascent transcription after exon 1, but not exon 7 siRNA, of *GNG12-AS1* were found using intronic probes (left panel). Both siRNAs decreased the number of exonic FISH signal (right panel). Cells positive for intronic probes include cells with both mono and biallelic signal, and cells positive for exonic probes include cells having one or more dots. The number of cells positive for RNA FISH signal is presented relative to control siRNA (control siRNA=454 cells; exon 1 siRNA=429 cells; exon 7 siRNA=257 cells). (**c**,**d**) siRNA-mediated knockdown of exon 1 (5′ targeting) and exon 7 of *GNG12-AS1* (3′ targeting) in HB2 (mammary epithelial cell line, **c**) and SUM159 (breast cancer cell line, **d**). Similar results were obtained in two additional cell lines (Supplementary Fig. 3a,b). *DIRAS3* is upregulated only when the 5' end of *GNG12-AS1* is targeted by siRNA directed to exon 1. For all the graphs, expression levels of *DIRAS3, GNG12, WLS, GNG12-AS1* were measured by qRT–PCR, normalized to *GAPDH* or to geometric mean of *GAPDH* and *RPS18* (see also Supplementary Fig. 3c–f), and are presented relative to control siRNA. Primers spanning exons 7–8 were used for *GNG12-AS1* expression. *RPS18* was used as a negative control gene whose expression does not change upon siRNA treatment. Error bars, s.e.m. (*n*=3 biological replicates). *P<0.05, **P<0.01, ***P<0.001 and ****P<0.0001 by two-tailed Student's *t*-test.

To investigate the role of *GNG12-AS1*, we depleted *GNG12-AS1* using siRNAs (Fig. 2a). *GNG12-AS1* has multiple isoforms^31^, thus we designed siRNAs against several different exons and found that siRNA targeted to exons 1, 5 and 7 reduced *GNG12-AS1* by 50–70% in HB2 and SUM159 cells (Supplementary Fig. 2a–c). These exons are common to all of the known splice forms of *GNG12-AS1*. Using several primer sets to capture the various *GNG12-AS1* isoforms, we confirmed that siRNAs against exons 1 and 7 efficiently knockdown *GNG12-AS1* (Supplementary Fig. 2d). We further confirmed that the knockdown occurred in all cellular compartments (Supplementary Fig. 2e). Thus, we are able to deplete *GNG12-AS1* in the nucleus with siRNA.

RNA FISH analysis in SUM159 cells showed that although siRNAs to either exon 1 or exon 7 effectively ablated *GNG12-AS1* detectable with exonic probes, siRNA targeting exon 1 reduced *GNG12-AS1* detectable with intronic probes (nascent transcripts) more efficiently than siRNA to exon 7 (Fig. 2b). These results indicate that although both siRNAs can efficiently reduce *GNG12-AS1* levels in different cellular compartments, siRNA complementary to the 5′ end of *GNG12-AS1* may preferentially inhibit its nascent transcription.

We next asked whether depletion of *GNG12-AS1* affects the transcription of its neighbouring genes. We analysed the expression of *GNG12*, *DIRAS3* and *WLS* after depleting *GNG12-AS1* with siRNAs against exon 1 and exon 7. *DIRAS3,* but not *GNG12* or *WLS,* was upregulated after targeting exon 1 of *GNG12-AS1* in four cell lines (Fig. 2c,d and Supplementary Fig. 3a–f). *DIRAS3* upregulation was consistent in different cell lines when *GAPDH* (Fig. 2c,d and Supplementary Fig. 3a,b) or the geometric mean of *GAPDH* and *RPS18* were used (Supplementary Fig. 3c–f). Thus, for further quantitative real-time PCR (qRT–PCR) experiments, we used *GAPDH* as the reference gene. In a fifth cell line MCF7, despite a 60% reduction in *GNG12-AS1,* expression *of DIRAS3* could not be reactivated (Supplementary Fig. 4a). In MCF7 cells, *DIRAS3* is more hypermethylated at its promoter compared with SUM159 cells^31^. Off-target effects were excluded by the use of additional siRNAs against exon 1 and exon 7 of *GNG12-AS1* (Supplementary Fig. 4b). Furthermore, we used the randomized nucleotide sequence (scrambled siRNAs) of exon 1 and exon 7 siRNAs (Supplementary Fig. 4c,d), as well as C911 mismatch siRNA controls^38^, all of which failed to affect *GNG12-AS1* and *DIRAS3* levels (Supplementary Fig. 4e).

To exclude the possibility of *GNG12-AS1* regulating *DIRAS3* expression in *trans*, we ectopically introduced *GNG12-AS1* in SUM159 and HB2 cells. Overexpression of the most common splice variants of *GNG12-AS1* had no effect on expression of *DIRAS3* or the surrounding genes (Supplementary Fig. 5a,b). As *DIRAS3* is an imprinted gene, we examined whether allele-specific expression changed after *GNG12-AS1* depletion. *DIRAS3* imprinting and its methylation status were unaltered (Supplementary Fig. 5c–e). Taken together, these results suggest that *GNG12-AS1* modulates the expression of the already active *DIRAS3* allele in *cis* and that siRNA to exon 1 of *GNG12-AS1* disrupts this function without affecting expression of neighbouring genes.

### siRNA against 5′ end of *GNG12-AS1* blocks RNA Pol II

Next, we examined whether inhibition of *DIRAS3* in *cis* can be achieved by targeting *GNG12-AS1* exons closer to the *DIRAS3* gene. Similar to when we targeted exon 7, siRNA to exon 5 efficiently reduced *GNG12-AS1* (Supplementary Fig. 2b,c), but did not upregulate *DIRAS3* (Supplementary Fig. 6a,b). In one of the cell lines, siRNA to exon 5 led to *WLS* downregulation. siRNAs to exons 2 and 3, which flank *DIRAS3* on either side, reduced *GNG12-AS1* isoform specifically (Supplementary Fig. 2f,g), suggesting that these siRNAs function post-transcriptionally. These siRNAs had no effect on *DIRAS3* expression further supporting modulation via the act of transcription rather than through a specific *GNG12-AS1* isoform (Supplementary Fig. 6c,d).

**Figure 3:**
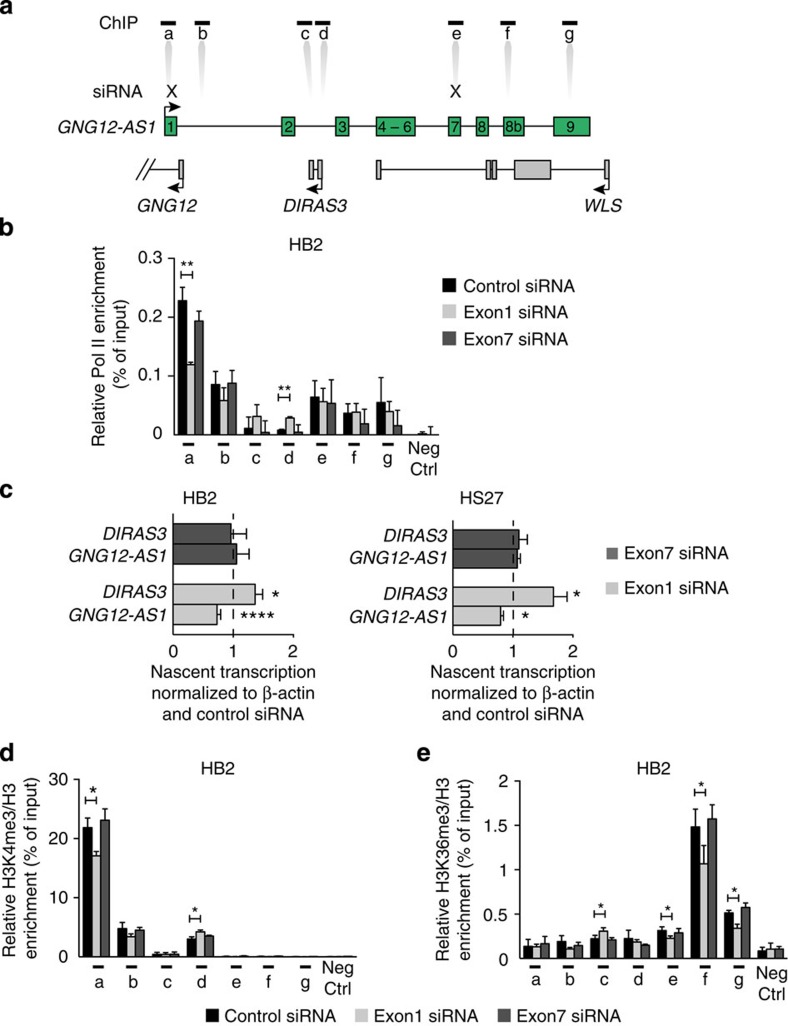
Inhibition of Pol II after siRNA targeted to exon 1 of *GNG12-AS1*. (**a**) The *GNG12-AS1* locus showing the location of primer sets (a–g) used for chromatin immunoprecipitation (ChIP) analysis. *GNG12-AS1* TSS (a), region 1.6 kb downstream of *GNG12-AS1* TSS (b), exon 2 of *DIRAS3* (c), *DIRAS3* TSS (d), *GNG12-AS1* exon 7 (e), *GNG12-AS1* exon 8b (f) and *GNG12-AS1* exon 9 (g). The X indicates the exons where siRNAs were targeted. (**b**) Pol II ChIP analysis in HB2 cells treated with siRNA to exons 1 or 7 and controls. ChIP enrichments are presented as the percentage of protein bound, normalized to input. siRNA targeting exon 1 of *GNG12-AS1*, but not exon 7, results in reduced Pol II binding at *GNG12-AS1* TSS (a). At the same time, increased Pol II binding was observed at the *DIRAS3* TSS (d). (**c**) Nuclear run-on analysis of *GNG12-AS1* and *DIRAS3* transcription after treatment of HB2 cells (left panel) or HS27 cells (right panel) with control and exon 1 or exon 7 *GNG12-AS1* siRNA. siRNA targeting exon 1 results in reduced *GNG12-AS1* nascent transcription, which leads to increased *DIRAS3* transcription. Data are normalized to *β-actin* and standardized to control siRNA, which is set up as 1 and shown as dotted line. (**d**,**e**) ChIP analysis from HB2 cells treated with siRNA to exons 1 or 7 using H3K4me3 (**d**) and H3K36me3 (**e**) antibodies. The precipitated DNA fragments were subjected to qRT–PCR analysis with the same primers as for Pol II ChIP. siRNA targeting exon 1, but not exon 7, of *GNG12-AS1* reduced H3K4me3 at *GNG12-AS1* TSS (a) and H3K36me3 levels in the body of *GNG12-AS1* (e–g). Concomitantly, H3K4me3 and H3K36me3 levels increased at the *DIRAS3* (d,c, respectively). Levels of H3K4me3 and H3K36me3 were normalized to histone H3 density. For all the graphs, ChIP enrichments are presented as the percentage of protein bound, normalized to input. Neg ctrl=negative control region. Error bars, s.e.m. (*n*=3 biological replicates). *P<0.05, **P<0.01 and ****P<0.0001 by two-tailed Student's *t*-test.

To prove that the *cis* function of *GNG12-AS1* is modulated by siRNA targeting exon 1, which leads to the inhibition of TI between *GNG12-AS1* and *DIRAS3*, we profiled Pol II and histone modifications. As shown in Fig. 3a,b, a significant reduction in Pol II binding was observed at the TSS of *GNG12-AS1* when exon 1, but not exon 7, was targeted by siRNA. Concomitantly, an increase in Pol II binding was observed at the *DIRAS3* TSS. Nuclear run-on assays showed that the reduction in Pol II binding was associated with reduced nascent *GNG12-AS1* transcription. Thus, a reduction of *GNG12-AS1* and increased *DIRAS3* run-on transcripts were observed with siRNA targeting exon 1, but not exon 7 (Fig. 3c).

To determine whether we could ablate unspliced transcripts and if so, whether this could affect *DIRAS3* expression, we designed siRNAs to the first intron at 195 and 2,933 bp downstream of the *GNG12-AS1* TSS. In both HB2 and HS27 cell lines, these siRNAs resulted in a reduction of *GNG12-AS1*, indicating that the unspliced *GNG12-AS1* transcript can be targeted by siRNA. However, increased *DIRAS3* expression occurred only in HS27 cells (Supplementary Fig. 7a,d), indicating that siRNA specifically targeting exon 1 is more efficient at disrupting the regulatory relationship between *GNG12-AS1* and *DIRAS3* transcription.

Consistent with the changes in Pol II binding, we observed changes in active histone modifications when *GNG12-AS1* was depleted using siRNA against exon 1, but not with exon 7 (Fig. 3d,e). Histone H3 lysine 4 trimethylation (H3K4me3) was reduced at the *GNG12-AS1* TSS and increased at the *DIRAS3* TSS. Furthermore, histone H3 lysine 36 trimethylation (H3K36me3), a marker for Pol II elongation^39^, was decreased in the body of *GNG12-AS1* and increased within *DIRAS3*. As *GNG12-AS1* depletion with exon 1 siRNA did not affect *WLS* expression, a reduction in H3K36me3 at 3′ end of *GNG12-AS1* correlates with reduction in its transcriptional activity. The reduction of active histone modifications at the *GNG12-AS1* TSS was not accompanied by a reciprocal increase of silencing modifications previously reported to be involved in TGS^20^,^21^,^29^, such as histone H3 lysine 9 trimethylation (H3K9me3) and histone H3 lysine 27 trimethylation (H3K27me3; Supplementary Fig. 8a,b). In addition, DNA methylation was unchanged at the *GNG12-AS1* TSS (Supplementary Fig. 8c).

The changes in Pol II binding at the TSS of *GNG12-AS1*, together with the changes in active histone modifications associated with transcriptional activity after siRNA to exon 1, suggest that targeting the 5′ end of this lncRNA results in transcriptional silencing. However, to date, in all instances of TGS with exogenous siRNAs in human cells, the siRNAs were directed to the gene promoters. We therefore designed two siRNAs targeting *GNG12-AS1* upstream of the TSS (33 and 129 bp upstream of TSS). These siRNAs had no effect on *GNG12-AS1* expression (Supplementary Fig. 7c,d), suggesting that transcription needs to be initiated for effective knockdown of the lncRNA.

In summary, siRNA targeting specifically exon 1 of *GNG12-AS1* reduced its nascent transcription, without inducing changes in silent chromatin marks. Nevertheless, reduced transcription was associated with diminished Pol II activity and the redistribution of active histone marks, which led to increased *DIRAS3* expression.

### AGO2 mediates transcriptional inhibition of *GNG12-AS1*

**Figure 4:**
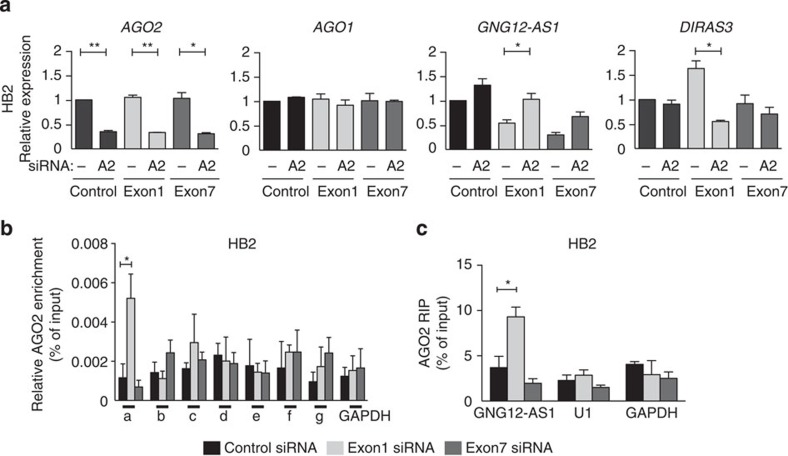
*AGO2* mediates transcriptional inhibition of *GNG12-AS1*. (**a**) HB2 cells were transfected with siRNA to exon 1 or exon 7 of *GNG12-AS1* together with control siRNA and siRNA targeting *AGO2* (A2) (**a**) or *AGO1* (A1) (Supplementary Fig. 9a). Expression levels of *AGO2, AGO1, DIRAS3* and *GNG12-AS1* were normalized to *GAPDH* and compared with control siRNA by qRT–PCR. *AGO1* levels were not affected by either single *AGO2* siRNA or double knockdown of *AGO2* and *GNG12-AS1*. Transcriptional upregulation of *DIRAS3* can be rescued by depletion of *AGO2* and siRNA targeting exon 1 of *GNG12-AS1,* whereas siRNA-mediated reduction of *GNG12-AS1* with both siRNAs expression can be rescued by depletion of AGO2. (**b**) AGO2 ChIP analysis in HB2 cells after siRNA depletion of *GNG12-AS1*. The *x* axis shows enrichment of AGO2 at regions described in Fig. 3a. AGO2 binding was enriched only at the TSS of *GNG12-AS1* (**a**) after siRNA to exon 1. The *GAPDH* TSS was used as negative control region for AGO2 ChIP. (**c**) RIP from nuclear HB2 extracts showing association of AGO2 with *GNG12-AS1* transcript after treatment of cells with exon 1 but not exon 7 siRNA. *U1* and *GAPDH* were used as negative control RNAs for AGO2 RIP. RIP enrichments are presented as % of input RNA. For all the graphs, error bars indicate s.e.m. (*n*=3 biological replicates). *P<0.05 and **P<0.01 by two-tailed Student's *t*-test.

As TGS is achieved through the RNAi machinery^24^,^25^,^27^,^28^, we investigated whether siRNA-mediated silencing of *GNG12-AS1* involves Argonaute proteins. First, we examined whether the reduction in *GNG12-AS1* transcript by siRNA targeted to exon 1 could be rescued by depletion of AGO1/2 proteins. We found that depletion of AGO2 rescued *GNG12-AS1* expression in cells exposed to siRNAs against exon 1 and exon 7 and abolished *DIRAS3* upregulation in cells treated with siRNA against exon 1 (Fig. 4a). This effect was specific to AGO2, as AGO1 depletion did not rescue *GNG12-AS1* depletion or *DIRAS3* levels (Supplementary Fig. 9a). Similar results were obtained in the HS27 cell line (Supplementary Fig. 9b,c), confirming that AGO2 is involved in both the transcription- and post-transcription-mediated mechanism of siRNA-directed downregulation of *GNG12-AS1.*

We verified that AGO2 is present in the nucleus and cytoplasm (Supplementary Fig. 9d), as previously reported^18^. Next, we used chromatin immunoprecipitation (ChIP) analysis to see whether AGO2 associates with the *GNG12-AS1* locus. We found that AGO2 was recruited to the *GNG12-AS1* TSS only after treatment with siRNA directed to exon 1, but not exon 7 (Fig. 4b). This result suggests that AGO2 facilitates the physical interaction between exogenous siRNA to exon 1 and the chromatin to potentially mediate TGS of *GNG12-AS1*. Finally, we tested whether *GNG12-AS1* associates with AGO2 in the nucleus. RNA immunoprecipitation (RIP) for AGO2 in nuclear extracts transfected with siRNAs showed an interaction between *GNG12-AS1* and AGO2 only when siRNA targeted exon 1, and not exon 7 (Fig. 4c). Taken together, these results demonstrate that AGO2 mediates transcriptional silencing of *GNG12-AS1* by siRNA complementary to exon 1.

### Cell cycle regulation by *GNG12-AS1*

**Figure 5:**
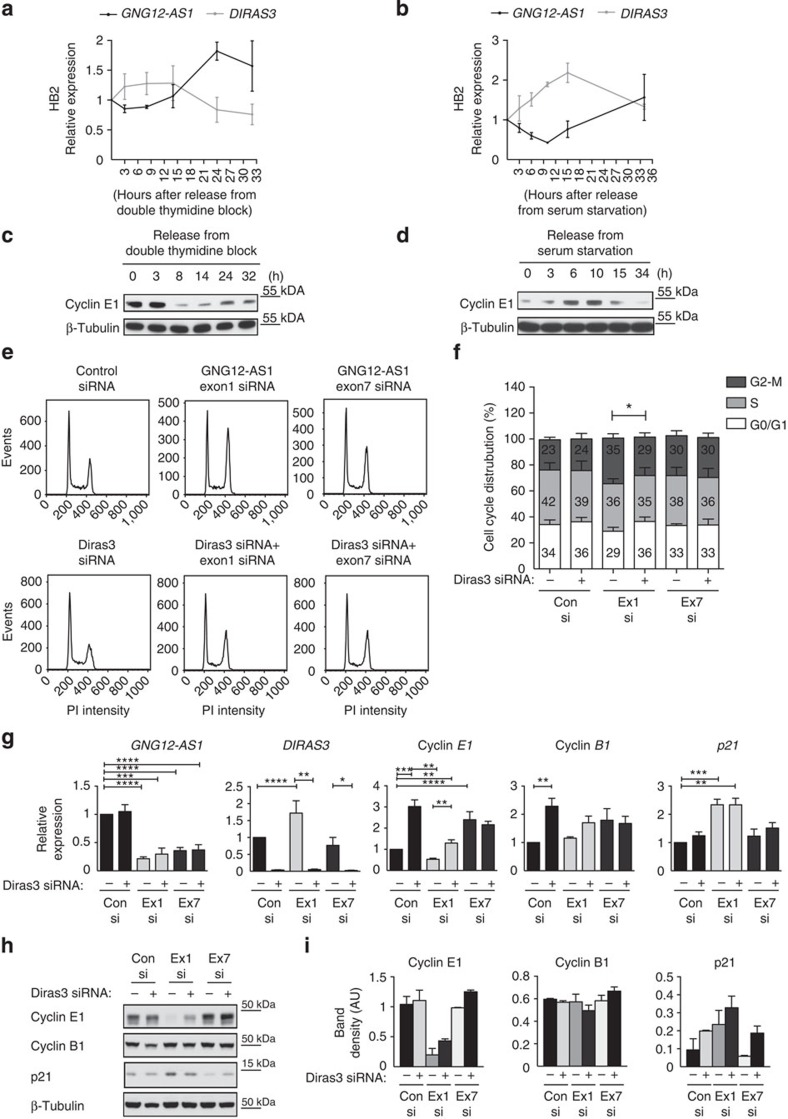
*DIRAS3*-dependent and independent cell cycle regulation. (**a**,**b**) Expression patterns of *GNG12-AS1* and *DIRAS3* are inversely correlated in synchronized cells. HB2 cells were synchronized by double thymidine block (**a**) or serum starvation (**b**) and released at indicated time points. Expression levels were measured by qRT–PCR, normalized to *GAPDH* and standardized to time point 0 h (set as 1). Error bars, s.e.m. (*n*=3 biological replicates). (**c**,**d**) Immunoblot of cyclin E1 (G1/S transition marker) was used to monitor cell cycle progression of HB2 cells synchronized with a double thymidine block (**c**) or serum starvation (**d**). β-Tubulin was used as a loading control. (**e**) FACS analysis of asynchronous HB2 cells after depletion of *GNG12-AS1*, *DIRAS3* or simultaneous depletion of *GNG12-AS1* and *DIRAS3*. The histograms are representative images of four biological replicates. (**f**) Cell cycle distribution of HB2 cells following siRNA conditions as shown in **e**. Note the restoration of G1 cell number between *GNG12-AS1* exon 1 siRNA-treated cells and simultaneous depletion of *GNG12-AS1* and *DIRAS3* (control siRNA: 34% versus exon 1 siRNA: 29%, *P<0.05 by two-tailed Student's *t*-test). Increase in G2/M population was observed between control and exon 1 siRNA (control siRNA: 23% versus exon 1 siRNA: 35%, ***P<0.001) and exon 7 siRNA (control siRNA: 23% versus exon 7 siRNA: 30%, *P<0.05 by two-tailed Student's *t*-test). (**g**) qRT–PCR of *DIRAS3, GNG12-AS1, cyclin E1, cyclin B1* and *p21* after *GNG12-AS1* and *DIRAS3* depletion as shown in **e**. Expression levels are normalized to *GAPDH* and presented relative to control siRNA. Error bars, s.e.m. (*n*=4 biological replicates). *P<0.05, **P<0.01, ***P<0.001 and ****P<0.0001 by two-tailed Student's *t*-test. (**h**) Immunoblot of cyclin E1, cyclin B1 and p21 after *GNG12-AS1* and *DIRAS3* depletion in HB2 cells. Cyclin E1 was reduced only in exon 1 siRNA-treated cells and partially rescued after simultaneous depletion of *GNG12-AS1* and *DIRAS3* (compare lane 3 to lane 4). β-Tubulin was used as a loading control. (**i**) Quantification of immunoblots from (**h**; *n*=2 biological replicates).

*DIRAS3* has been implicated in cell cycle regulation^40^, so we next asked if expression levels of *DIRAS3* and *GNG12-AS1* might correlate at different stages of the cell cycle. We synchronized cells with a double thymidine block (Fig. 5a), which arrest cells in S-phase and used cyclin E1 (G1/S transition marker) to monitor cell cycle progression (Fig. 5c). An increase in *GNG12-AS1* transcription was observed after the release from double thymidine block, which coincided with the downregulation of *DIRAS3.* Cell cycle synchronization using serum starvation, followed by release with serum-containing media, showed similar results (Fig. 5b,d). The inverse relationship between expression levels of *GNG12-AS1* and *DIRAS3*, which was tightly maintained throughout the cell cycle, further supports the evidence that *GNG12-AS1* transcription modulates *DIRAS3* expression in *cis* through TI.

We next examined the consequence of upregulating *DIRAS3* after inhibiting *GNG12-AS1.* When *GNG12-AS1* was depleted with exon 1 siRNA, we observed a small decrease in G1 cell number (control siRNA: 34% versus exon 1 siRNA: 29%; *P*=0.0575; Fig. 5e,f). This result was confirmed by qRT–PCR and immunoblot analysis, which showed decreased *cyclin E1* levels (Fig. 5g–i). As expected, by combining siRNAs to *DIRAS3* and exon 1 siRNA, we were able to neutralize the increase in *DIRAS3* expression (Fig. 5g–i), which led to a modest but significant restoration of the G1 population (exon 1 siRNA: 29% versus exon 1/DIRAS3 siRNA: 36%, *P*=0.0105; Fig. 5f). Analysis of *cyclin E1* levels showed that the reduction in G1 cell numbers could be partially rescued with the simultaneous depletion of *GNG12-AS1* and *DIRAS3* (Fig. 5g–i). By contrast, *cyclin B1* levels did not differ among *GNG12-AS1-* and *DIRAS3* siRNA-treated cells. Both RNA and protein levels of the cell cycle inhibitor *p21* increased when the cells were treated with siRNA to exon 1 in a *DIRAS3-* independent manner. S-phase distribution was unchanged after reducing *GNG12-AS1*. However, both exon 1 and exon 7 siRNAs increased the G2/M population (control siRNA: 23% versus exon 1 siRNA: 35%, *P*=0.0009; or versus exon 7 siRNA: 30%, *P*=0.0126). As the increase in G2/M population occurred upon simultaneous depletion of *GNG12-AS1* and *DIRAS3* (control siRNA: 23% versus exon 1/DIRAS3 siRNA: 29%, *P*=0.0169; or versus exon 7/DIRAS3 siRNA: 30%, *P*=0.0084), we concluded that this G2/M delay is a *DIRAS3*-independent event.

Taken together, these data indicate that *GNG12-AS1* has a dual function in controlling cell cycle progression. Transcription of *GNG12-AS1* influences the G1 phase of the cell cycle by modulating *DIRAS3* expression, whereas the effect on G2/M phase of the cell cycle by *GNG12-AS1* is a *DIRAS3-*independent event, and most likely represents the function of the RNA product.

### *GNG12-AS1* has a *trans* function regulating cell migration

**Figure 6:**
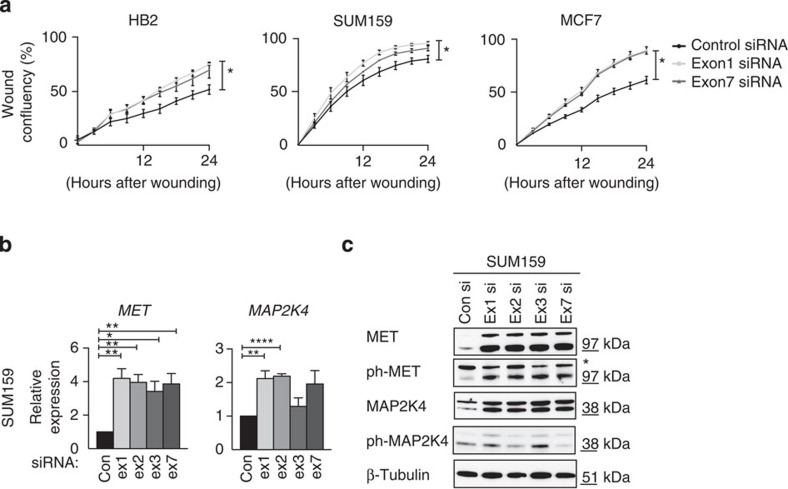
*GNG12-AS1* regulates cell migration independently of *DIRAS3*. (**a**) Cell migration is increased in HB2, SUM159 and MCF7 cells after *GNG12-AS1* depletion. Cells were treated with control, exon 1 and exon 7 *GNG12-AS1* siRNAs for 48 h before the wound-healing assay was performed over 24 h time course. Cells depleted with siRNA targeted to exon 1 and exon 7 of *GNG12-AS1* have significantly increased cell migration. (**b**) Validation of gene expression changes in SUM159 cells after depletion of *GNG12-AS1* with siRNAs targeting exons 1 and 7. Expression levels of *MET* and *MAP2K4* were measured by qRT–PCR, normalized to *GAPDH* and compared with control siRNA. The knockdown efficiency of *GNG12-AS1* is shown in Supplementary Fig. 2b,f. (**c**) Immunoblot of MET, MAP2K4 and their active phosphorylated (ph) forms in SUM159 cells after depletion of *GNG12-AS1* with siRNAs targeting exons 1, 2, 3 and 7. Asterisk (*) indicates a non-specific band detected with an antibody specific to phosphorylated MET. β-Tubulin was used as a loading control. For all the graphs, error bars indicate s.e.m. (*n*=3 biological replicates). *P<0.05, **P<0.01 and ****P<0.0001 by two-tailed Student's *t*-test.

*DIRAS3* overexpression has been linked to cell migration and cell proliferation^32^, so we assayed these cellular phenotypes after *GNG12-AS1* knockdown. We found that reducing *GNG12-AS1* with either of the siRNAs increased cell migration (Fig. 6a). As no difference was seen between the two siRNAs, we conclude that these phenotypic effects are due to the reduction of *GNG12-AS1* transcripts and independent of *DIRAS3*. Indeed, a change in the migration phenotype was also observed in MCF7 cells, where *DIRAS3* silencing cannot be reversed by *GNG12-AS1* depletion (Fig. 6a). We next profiled global transcription changes after knockdown of *GNG12-AS1* and found that both siRNAs against this lncRNA induced significant changes in cell adhesion and actin cytoskeleton pathways (Supplementary Fig. 10a,b). We focused on mesenchymal epithelial transition factor (MET), a tyrosine kinase receptor for the hepatocyte growth factor, known to regulate cell migration and invasion^41^,^42^ and found increased levels of MET (Fig. 6b,c and Supplementary Fig. 10c). In line with MET activation, we found elevated levels of the *MET* downstream target *MAP2K4* (mitogen-activated protein kinase kinase 4)^42^. This suggests that activation of MET signalling and its downstream targets could be responsible for the observed changes in cell migration. Interestingly, reducing specific minor isoforms of *GNG12-AS1* containing exons 2 and 3 was sufficient to trigger activation of this signalling pathway. Future research into the secondary structure of the *GNG12-AS1* will determine if this function is isoform specific. Finally, we examined whether simultaneously depleting *GNG12-AS1* and *DIRAS3* would prevent MET activation. Increased MET protein levels were maintained, confirming that *GNG12-AS1* regulates MET signalling independently of *DIRAS3* (Supplementary Fig. 10d). Altogether, these data indicate that the *GNG12-AS1* transcript inhibits cell migration. In this context, *GNG12-AS1* may have a tumour-suppressor function in addition to a role in modulating the expression levels of *DIRAS3.*

## Discussion

LncRNAs can function via their RNA product and through the act of their transcription. So far it has been challenging to tease apart these two processes. Here we employed an siRNA-based strategy targeting different regions of *GNG12-AS1* and found that siRNA selectively targeting the first exon of *GNG12-AS1* resulted in increased expression of *DIRAS3* in *cis*. These findings, together with inverse relationship between *GNG12-AS1* and *DIRAS3* during the cell cycle and Actinomycin D treatment, indicate that the act of transcribing *GNG12-AS1* regulates expression of the active *DIRAS3* allele by TI. We subsequently showed that this transcriptional targeting by siRNA to the 5′ end of *GNG12-AS1* is mediated by AGO2, which binds to the *GNG12-AS1* TSS as well as to its transcript. Finally, distinct functional consequences were uncovered, depending on whether *GNG12-AS1* transcription was disrupted or whether its product was depleted.

The first suggestion that siRNA targeting exon 1 of *GNG12-AS1* reduced its nascent transcription was with RNA-FISH, where we observed reduced hybridization with intronic probes after treating cells with this siRNA. More concrete biochemical evidence of transcriptional inhibition were demonstrated by decreased Pol II binding at the *GNG12-AS1* TSS with ChIP and reduced nascent transcription with nuclear run-on experiments. siRNA targeted to exon 7, while still reducing the overall *GNG12-AS1* product, does not upregulate *DIRAS3* and does not change Pol II binding, making this siRNA an excellent internal control for our experiments.

Consistent with the data on Pol II binding and nascent transcription, we detected a reduction in the active histone modification, H3K4me3 at the *GNG12-AS1* TSS when *GNG12-AS1* was depleted using siRNA against exon 1, but not with exon 7. Similarly, the histone modification mark H3K36me3, which normally accumulates in gene body where it is associated with transcriptional elongation^39^, was reduced at the 3′ end of *GNG12-AS1*. Thus, our findings suggest that siRNA targeting the 5′ end of *GNG12-AS1* led to inhibition of transcriptional initiation and elongation of *GNG12-AS1* by directly interfering with Pol II dynamics without inducing changes in DNA methylation and repressive histone marks.

To date, all reported cases of exogenous TGS in human cells have used siRNAs against the gene promoter regions or the TSS^17^,^20^,^24^,^25^,^29^. Our study is, therefore, the first to show that targeting the first exon interferes with the transcription of a lncRNA. Indeed, with *GNG12-AS1*, targeting regions upstream of the TSS had no effect on its transcription, suggesting that transcription needs to be initiated in order to be targeted by siRNA. This may also explain why, although we found a reduction in active histone modifications, we did not observe an increase in repressive chromatin marks.

As TGS is achieved through the RNAi machinery^24^,^25^,^27^,^28^, we investigated the engagement of AGO2 in the inhibition of *GNG12-AS1* transcription. siRNAs are known to form a complex with AGO2 in the cytoplasm and then shuffle between the cytoplasm and nucleus^43^. Small-RNA loading is a cytoplasmic process necessary for the nuclear import of AGO2 (refs 18, 43, 44). It has further been suggested that small RNAs can guide AGO2 to the promoters of ncRNAs and affect their transcription^45^,^46^. Based on these precedents, we would expect that all siRNAs in our experiments would have a similar knockdown efficiency in different cellular compartments, and once the AGO2–siRNA complex was in the nucleus, the functional consequences of the *GNG12-AS1* depletion would depend on the region targeted. The presence of AGO2–siRNA complexes in the nucleus and cytoplasm would explain why we could rescue the *GNG12-AS1* expression in double knockdown experiments between *GNG12-AS1* and AGO2. However, AGO2-siRNA-*GNG12-AS1* complexes at the *GNG12-AS1* TSS could only be detected in the nucleus when the siRNA was directed to exon 1.

**Figure 7:**
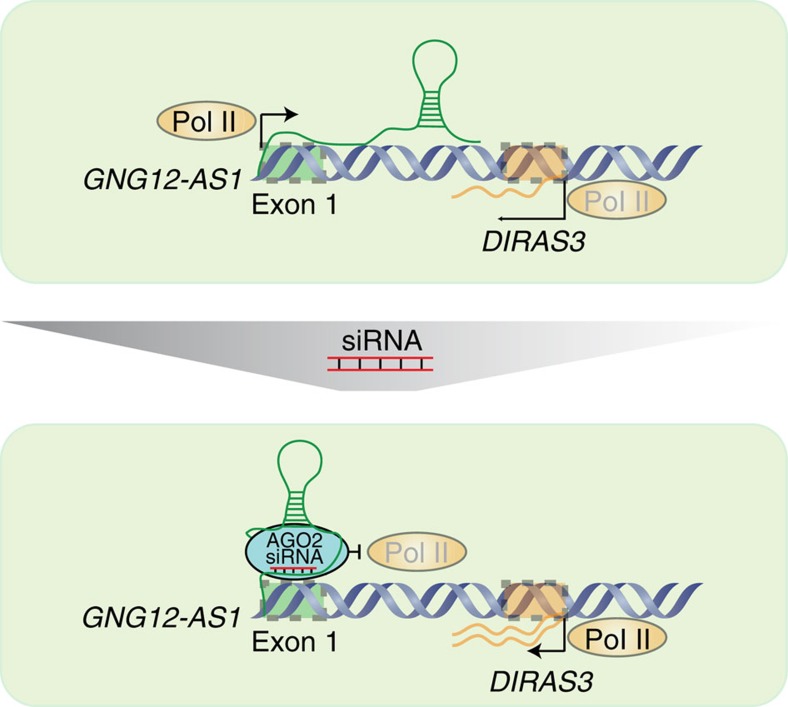
Inhibition of transcriptional interference with siRNA. The top panel depicts how *GNG12-AS1* modulates the expression of the active *DIRAS3* allele through transcriptional interference. *GNG12-AS1* may act as a rheostat for *DIRAS3* transcription rate. The lower panel depicts how exogenous siRNA molecules in a complex with AGO2 can bind to both the TSS and *GNG12-AS1* to inhibit Pol II, and block further transcription initiation and elongation of *GNG12-AS1*. As a result of transcriptional silencing *of GNG12-AS1,* transcriptional interference is reduced leading to increased transcription of *DIRAS3*.

As described above, *GNG12-AS1* could only be reduced when sequences downstream of its TSS were targeted by siRNA. This finding, together with the RIP results, showing that AGO2 associates with the *GNG12-AS1* when cells are treated with siRNA to exon 1, suggest that transcription needs to have been already initiated for siRNA-mediated repression of transcription. Although AGO2–siRNA interactions with chromatin and the lncRNA could be demonstrated by ChIP and RIP, respectively, we do not have direct evidence for siRNA–AGO2 attaching the nascent lncRNA to the chromatin. We propose a model described in Fig. 7, where exogenous siRNA complementary to the 5′ end (exon 1) of *GNG12-AS1* recruits AGO2 to *GNG12-AS1* and to the chromatin at its TSS. This complex, containing the siRNA and *GNG12-AS1* and possibly other proteins, stalls transcription of *GNG12-AS1* by Pol II. *DIRAS3* expression is enhanced as a consequence of reduced *GNG12-AS1* transcription.

There are two possible mechanisms whereby an exogenous siRNA in a complex with AGO2 could lead to decreased *GNG12-AS1* transcription. AGO2 has slicer activity^47^ and this could promote cleavage and degradation of the nascent *GNG12-AS1.* Alternatively, the AGO2–siRNA complex could present a physical block for Pol II initiation and elongation at the *GNG12-AS1* TSS. Indeed, AGO2 has been shown to guide small RNAs to inhibit Pol II recruitment and elongation in human cells^24^,^48^ independently of its slicer activity^24^,^27^,^45^,^46^. AGO2 has been reported to form a complex with different chromatin-modifying proteins to mediate gene silencing^49^. It is therefore possible that AGO2–siRNA–*GNG12-AS1* complexes at the TSS may include further proteins that influence Pol II kinetics. Our model does not distinguish between these different but not necessarily mutually exclusive mechanisms.

Silencing of imprinted genes is frequently achieved by TI of an antisense lncRNA^11^,^50^. In these cases, the lncRNA is reciprocally imprinted relative to its target gene and is expressed on the opposite allele. Unlike, the other imprinted genes, we found that it was the already actively expressing *DIRAS3* allele being upregulated after *GNG12-AS1* knockdown, indicating that *GNG12-AS1* has a role in modulating *DIRAS3* expression in *cis*, rather than maintaining its imprinting. A reduction of Pol II and H3K4me3 at the TSS of *GNG12-AS1* was associated with a concomitant increase at the *DIRAS3* TSS. These shifts reflect the change in dynamics between these two genes as transcription inhibition by the *GNG12-AS1* is eased and *DIRAS3* transcription is augmented. TI between *GNG12-AS1* and *DIRAS3* could only be consistently inhibited when siRNAs were target within the first exon of *GNG12-AS1*. Targeting the adjacent intron did not lead to *DIRAS3* upregulation in both of the cell lines tested. As *GNG12-AS1* was efficiently reduced, we presume the nascent transcript was targeted, but *GNG12-AS1* may also be present as mature unspliced transcripts that can be targeted post-transcriptionally. There are several enhancer signature peaks (histone H3 lysine 4 monomethylation (H3K4me1) and histone H3 lysine 27 acetylation (H3K27ac)) within this intron as annotated in the ENCODE database (Supplementary Fig. 11). It is possible that although the nascent RNA can be inhibited by siRNA targeted to this region, enough transcription has already occurred through the region to prevent *DIRAS3* upregulation. In a region with multiple enhancers, some are likely to be tissue specific, which could explain why *DIRAS3* expression is differentially upregulated in two different cell lines. The presence of enhancer marks at this locus would fit with *GNG12-AS1* being an enhancer-like, *cis* regulator of *DIRAS3* transcription^51^. The location of these enhancers and their potential specificity for *DIRAS3* might explain why *GNG12* is not affected by *GNG12-AS1* depletion. Another possibility is that *GNG12-AS1* expression is limited by *GNG12* transcription and its further reduction has no impact on *GNG12* expression.

An alternative strategy to siRNA for analysing the *cis* functions of *GNG12-AS1* would have been to use CRISPRi, a new technology involving small guide RNAs to recruit a catalytically inactive Cas9 (dCas9) fused to the KRAB transcriptional repressor domain to specific loci^52^. Similar to RNAi, CRISPRi does not affect the underlying gene sequence and was shown to induce transcriptional repression of lncRNAs^52^. However, KRAB-mediated silencing can spread to adjacent genes^53^, which would confound the detection of genes influenced by TI by a lncRNA.

Transcription of lncRNA provides an important mechanism for fine-tuning the expression of genes in *cis*^54^. There is evidence for lncRNAs having a threshold-depended role in moderating gene regulation rather than an activating or repressive role^54^. For example, *MALAT1,* a lncRNA with several roles in gene regulation, has subtle effects on the expression of its neighbouring genes^55^. A fine-tuning mechanism could explain why small changes in gene expression observed after depletion of some lncRNAs could be relevant upon specific stress or environmental conditions. Imprinted genes are exquisitely regulated for gene dosage. It is likely that *in vivo*, the *cis* regulatory function of *GNG12-AS1* in fine-tuning *DIRAS3* expression is limited to specific developmental stages or environmental conditions as shown for *HOTTIP* lncRNA^56^, which has similarly low abundance and expression levels. *GNG12-AS1* is not imprinted in non-cancer cell lines, as shown previously by pyrosequencing SNP analysis^31^, but our RNA-FISH results indicate that in a substantial proportion of HB2 cells it may be randomly monoallelic. A growing number of autosomal genes are being shown to be randomly monoallelic at single-cell level^57^. If 50% of cells with *GNG12-AS1* expression are expressing transcripts from the paternal allele, these would be repressing *DIRAS3* in *cis* by TI, whereas on the other 50% of cells where *GNG12-AS1* expression is maternal, *DIRAS3* would already be silent on this allele. This would be consistent with the suggestion that random monoallelic expression refines gene expression levels in a population of cells^58^,^59^.

In addition to *GNG12-AS1* having a *cis* function in moderating *DIRAS3* transcription, *GNG12-AS1* also regulates cell cycle progression. Transcriptional upregulation of *DIRAS3* induced by *GNG12-AS1* depletion led to G1 delay associated with the downregulation of *cyclin E1*. As the G1 delay and *cyclin E1* levels were rescued by simultaneous depletion of exon 1 siRNA of *GNG12-AS1* and *DIRAS3*, these data suggest that TI between *GNG12-AS1* and *DIRAS3* contributes to the regulation of G1 phase of the cell cycle. *DIRAS3* was shown to be involved in cell cycle progression^40^,^59^. The upregulation of *DIRAS3* by inhibiting *GNG12-AS1* transcription in our study does not have the same effect on cyclin genes as previously reported, where *DIRAS3* was ectopically overexpressed^40^,^59^,^60^. These differences could be due to the modest upregulation of *DIRAS3* induced by transient repression of *GNG12-AS1* with siRNA, whereas the latter study ectopically expressed *DIRAS3.* Nevertheless, our results are consistent with a role for *DIRAS3* in cell cycle progression through G1 phase^40^,^59^. More importantly, these results indicate that disruption of TI between *GNG12-AS1* and *DIRAS3* can have effects on the cell cycle.

RNA-FISH analysis indicated that, in addition to co-localization between intronic and exonic probes, we also found cells in which the signals were separated, suggesting that there may be additional functions for *GNG12-AS1*. Our cell cycle analysis revealed that *GNG12-AS1* depletion with siRNAs to 5′ end and 3′ end resulted in a G2/M delay. This effect is most likely due to a *DIRAS3*-independent function of *GNG12-AS1* RNA product. This is further supported by our microarray experiments where both siRNAs induced changes in genes involved in cell cycle progression.

In addition, we found that reducing the levels of *GNG12-AS1* transcripts whether by siRNAs directed to exon 1 or various other exons of *GNG12-AS1* resulted in increased cell migration. This phenotype was observed upon *GNG12-AS1* knockdown even in cells where *DIRAS3* expression was absent. Expression profiling of *GNG12-AS1*-depleted cells showed an enrichment for genes involved in cell adhesion, migration and actin cytoskeleton pathways. We were able to validate the upregulation of MET signalling pathway after siRNA-mediated reduction of *GNG12-AS1*. MET controls many biological functions such as cell migration, invasion and motility, and its aberrant activation can lead to cancer^41^,^42^,^61^. We have previously shown that *GNG12-AS1* is downregulated in breast cancer tumours together with *DIRAS3* (ref. 31). Thus, it is possible that *GNG12-AS1* extends the tumour-suppressor function of this locus by regulating oncogenes such as MET. In keeping with this, MET was recently shown to be regulated by *MEG3* lncRNA in pancreatic tumours^62^. Thus, MET could be a common target of lncRNAs involved in the regulation of cellular processes important for tumour growth. Further experiments are necessary to investigate whether the *GNG12-AS1* transcript directly interacts with distant genes, and if so whether this interaction is isoform specific.

In summary, *GNG12-AS1* is similar to several other lncRNAs that have both *cis* and *trans* functions^63^,^64^. Although the *trans* function may be to regulate genes involved in cell cycle and cell migration through direct interaction, the *cis* function has a role in fine-tuning regulation of *DIRAS3* transcription. Altogether, our results demonstrate that an siRNA-based strategy can be employed to successfully separate functions that are due to lncRNA transcription from those of the transcript.

## Methods

### Cell culture and treatments

HB2 (human mammary epithelial cell line) were cultured in DMEM (GIBCO) supplemented with 10% fetal bovine serum (FBS), 10 ml l^−1^ penicillin–streptomycin solution, 5 mg ml^−1^ insulin (Sigma) and 1 mg ml^−1^ hydrocortisone (Sigma). MCF10A (human breast epithelial cell line) were cultured in MEGM SingleQuots (Lonza). SUM159 (breast cancer cell line) were cultured in Ham's F-12 medium (GIBCO) supplemented with 5% FBS, 5 mg ml^−1^ insulin and 1 mg ml^−1^ hydrocortisone; MCF7 (breast cancer cell line) and HS27 (human foreskin fibroblasts) were cultured in DMEM with 10% FBS and 10 ml l^−1^ penicillin–streptomycin solution. 293FT cells were grown in DMEM (high glucose 1 × ; GIBCO, ref. 41965039) supplemented with 10% FBS, 0.1 mM MEM Non-Essential Amino Acids (GIBCO), 6 mM L-glutamine (GIBCO), 1 mM MEM Sodium Pyruvate (GIBCO) and 500 μg ml^−1^ Geneticin (GIBCO). All the cells were from American Type Culture Collection and were cultured at 37 °C with 5% CO_2_. The cells were treated with Actinomycin-D (Sigma) at a final concentration of 10 μg ml^−1^ for 0, 2, 6, 8 and 24 h.

### Single-molecule RNA FISH

RNA FISH was performed as described^37^. Cells were grown on 18 mm round #1 cover glass, briefly washed with PBS and fixed with PBS/3.7% formaldehyde at room temperature for 10 min. Following fixation, cells were washed two times with PBS. The cells were then permeabilized in 70% ethanol for at least 1 h at 4 °C. Stored cells were briefly rehydrated with Wash Buffer (2 × saline sodium citrate buffer (SSC), 10% formamide) before FISH. The Stellaris FISH Probes (*GNG12-AS1* intronic (Q670) and exonic probes (Q570), sequences in Supplementary Table 1) were added to the hybridization buffer (2 × SSC, 10% formamide, 10% dextran sulfate) at the same time to give a final concentration of 250 nM per probe set. Hybridization was carried out in a humidified chamber at 37 °C overnight. The following day, the cells were washed twice with Wash Buffer at 37 °C for 30 min each. The second wash contained 4,6-diamidino-2-phenylindole for nuclear staining (5 ng ml^−1^). The cells were then briefly washed with 2 × SSC and then mounted in Vectashield (Vector Laboratories, H-1000). Images were captured using Nikon TE-2000 inverted microscope with NIS-elements software using a Plan Apochromat × 100 objective and Andor Neo 5.5 sCMOS camera. We acquired 25 optical slices at 0.3 μm intervals. Images were deconvolved with Huygens Professional and projected in two dimensions using Volocity Image Software Analysis. Intronic signals were scored to determine the percentage of cells with mono- or biallelic expression. Exonic signals were then scored on the same cells. To score whether intronic and exonic signals co-localize, we selected cells in which both signals were present.

### Quantitative real-time PCR

RNA (1 μg) was extracted with the RNeasy Kit (QIAGEN) and treated with DNase I (QIAGEN) following the manufacturer's instructions. QuantiTect Reverse Transcription Kit (QIAGEN) was used for cDNA synthesis including an additional step to eliminate genomic DNA contamination. Quantitative real-time PCR was performed on a 7900HT Fast Real-Time PCR System (Applied Biosystems) with Fast SYBR Green Master Mix (Life Technologies). Thermocycling parameters: 95 °C for 20 s followed by 40 cycles of 95 °C for 1 s and 60 °C for 20 s. Two reference genes (*GAPDH* and *RPS18*) were selected. Our microarray data show no significant variation in *GAPDH* and *RPS18* expression in our siRNA experiments in HB2, HS27 and SUM159 cells. Expression levels of *GNG12-AS1*, *DIRAS3, GNG12* and *WLS* in non-cancer and cancer cell lines are normalized relative to the geometric mean of *GAPDH* and *RPS18* (Supplementary Fig. 1a). The expression levels using either *GAPDH* (Fig. 2c,d and Supplementary Fig. 3a,b) or the average of *GAPDH* and *RPS18* (Supplementary Fig. 3c–f) across siRNA conditions were consistent and reproducible. Thus, we used only *GAPDH* normalization for further experiments. Exon 7–8 of *GNG12-AS1* was used to quantify its expression, if not stated otherwise. To calculate the *GNG12-AS1* copy number, standard curve of Ct values was performed by qRT–PCR using dilution series of known concentration of *GNG12-AS1* DNA template (variant 1 that contains exon 1, 2, 3, 4, 5, 6, 7, 8, 8b and 9). cDNA was made from RNA extracted from known number of different cell lines. The Ct values were fitted on the standard curve and the number of *GNG12-AS1* molecules per cell was calculated. The final value was multiplied by 2, to account for the fact that cDNA is single stranded and DNA templates used to make standard curve is double stranded. The sequences for expression primers are listed in Supplementary Table 2. The amplification efficiency of housekeeping genes was measured with serial dilution of cDNA of each gene from five different cell lines. PCR efficiencies and correlation coefficients (R2) for each primer pair are shown in Supplementary Table 3.

### RNA interference

The sequences of siRNAs are present in the Supplementary Table 4. The cells were transfected with DharmaFECT 1 (SUM159, MCF7, MCF10A), DharmaFECT 3 (HS27) or DharmaFECT 4 (HB2) (Thermo-Scientific) following the manufacturer's instructions. All experiments were done 48 h after transfection and all the siRNAs were used at a final concentration of 50 nM.

### siRNA-mediated depletion of *GNG12-AS1*

RNA and proteins were fractionated as described previously^65^. The cells were either untreated (for immunoblot analysis) or transfected with control and *GNG12-AS1* siRNAs (for RNA analysis). RNA was isolated from cytoplasmic, nucleoplasmic and chromatin fractions by TRIZOL extraction (Life Technologies) and used for qRT–PCR. Data were normalized to the geometric mean of *GAPDH* and *β-actin* levels in each cellular compartment as no normalization controls is equal in all three compartments (for details see ref. 65). *MALAT1* and *RPS18* were used as positive controls for chromatin and cytoplasmic fractions, respectively. Primers used for this assay are listed in Supplementary Table 2.

### Nuclear run-on assay

Nuclear run-on assay was performed as described previously^66^. Cells were grown in 10 cm dishes, trypsinized and centrifuged, and the pellets were washed with 1.5 ml of NP-40 lysis buffer (10 mM Tris-HCl, 10 mM NaCl, 3 mM MgCl_2_, 0.5% NP-40), incubated on ice for 5 min and centrifuged at 1,500*g* for 5 min. Pellets were then washed again with 1.5 ml NP-40 lysis buffer. The nuclei pellets were resuspended in 70 μl glycerol storage buffer (50 mM Tris-HCl, pH 8, 0.1 mM EDTA, 5 mM MgCl_2_, 40% glycerol) and flash frozen in liquid nitrogen. 60 μl were transferred to fresh nuclease-free tubes and equal amount of the 2 × run-on transcription buffer (20 mM Tris-HCl, pH8; 5 mM MgCl_2_, 300 mM KCl, 4 mM dithiothreitol) was added together with 2 mM ATP, CTP, GTP (GE Healthcare) and 1 mM Biotin-16-UTP (Epicentre). The reaction was incubated at 30 °C for 45 min. *In vitro* transcription was stopped by adding TURBO DNase (Ambion) at 37 °C for 30 min. Then 100 μl of nuclei lysis buffer (50 mM Tris-HCl, pH 7.5; 5% SDS, 0.125 M EDTA) and Proteinase K (Ambion) were added to the sample and incubated at 37 °C for 30 min. RNA extraction was performed using phenol/chloroform/isoamyl alcohol: 25:24:1 (Invitrogen) and chloroform/isoamyl alcohol: 24:1 (Sigma). To precipitate RNA, 3 M sodium acetate pH 5.5 (Life Technologies), GlycoBlue (Life Technologies) and ice-cold 100% ethanol were added. The samples were incubated at −80 °C for 30 min and centrifuged at 12,000*g* for 15 min. Pellets were washed with ice-cold 70% ethanol and centrifuged at 12,000*g* for 10 min. The pellets were then resuspended in 30 μl nuclease-free water. RNA samples were then purified on column using RNeasy kit (Qiagen) to remove free Biotin-16-UTPs, according to the manufacturer's instructions. Purified RNA from column was eluted in 30 μl nuclease-free water. Biotinylated RNA from samples was captured using Dynabeads MyOne C1 streptavidin beads (Invitrogen). C1 beads (40 μl per sample) were washed twice with Solution A (100 mM NaOH, 50 mM NaCl) for 3 min and once with Solution B (100 mM NaCl). The C1 beads were then preblocked with BSA (200 μg ml^−1^) and Yeast tRNA (200 μg ml^−1^; Invitrogen) mix in Solution B. The beads were pre-blocked on a rotor at room temperature for 30 min, collected on magnetic stand and resuspended in Solution B. Beads were aliquoted into nuclease-free tubes and equal amount (20 μl) of RNA was added. Biotinylated RNA was pulled down for 2 h at room temperature. Then the beads were collected on magnetic stand, washed three times with 1 × Wash/Binding buffer (2 × Wash/Binding buffer: 10 mM Tris-HCl, pH 7.4; 1 mM EDTA, 2 M NaCl). After the final wash, C1 beads with bound RNA were eluted in 30 μl nuclease-free water and used directly for reverse transcription to prepare cDNA. Relative transcription was calculated as: 2̂ −[MeanCt(gene)-MeanCt(β-actin)]. For *GNG12-AS1* knockdown experiments, control transcription was set to 1. *β-Actin* primer was used for normalization (primers are listed in Supplementary Table 2).

### Lentivirus overexpression of *GNG12-AS1* in human cells

The different *GNG12-AS1* splice variants and negative control vector (scrambled sequence) were first cloned in pJET1.2 plasmid (Fermentas) and then into modified pLenti6.3/TO/V5-DEST vector (kindly provided by John Rinn, Harvard University) using Gateway cloning strategy. The sequences are listed in Supplementary Data 1. The modified vector does not contain WPRE, the SV40 promoter and the blasticidin-resistance gene that could interfere with lncRNA structure and function. Sanger sequencing and restriction digestion using PvuI-HF and BsrGI enzymes confirmed the *GNG12-AS1* inserts. Lentiviral transduction of *GNG12-AS1* clones was done in 293FT cells using ViraPower (Invitrogen) including, negative and positive control vector containing mCherry. The DNA-Lipofectamine2000 complexes were added to 293FT cells and incubated overnight. Forty-eight and seventy-two hours post transfection, the virus-containing supernatants were harvested, centrifuged at 700*g* for 5 min at 4 °C and filtered with 0.45 μm with additional 0.22 μm filter before being stored at 4 °C. To transduce SUM159 cells, 80,000 cells per 12-well plate were seeded in triplicate and transduced with the lentiviruses at a multiplicity of infection of 0.1 together with polybrene (5 μg ml^−1^, Sigma). Transduction efficiency (>80%) was verified using positive control vector containing mCherry and measured by FACS Calibur Influx (Beckton Dickinson) using BDFACS Software 1.0.0.650. The FACS data were analysed using FlowJo software (TreeStar Inc). Exon 7–9 of *GNG12-AS1* was used to quantify its overexpression (primers are listed in Supplementary Table 2).

### Microarray analysis

Gene expression analysis was carried out on Illumina Human HT12 version 4 arrays. All data analyses were carried out on R using Bioconductor packages^67^. Raw intensity data from the array scanner were processed using the BASH^68^ and HULK algorithms as implemented in the bead array package^69^. Log2 transformation and quantile normalization of the data were performed across all sample groups. Differential expression analysis was carried out using the Limma package^70^. Differentially expressed genes were selected using a *P*-value cutoff of <0.05 after application of FDR correction for multiple testing applied globally to correct for multiple contrasts. RNA was extracted from cells (HB2, SUM159) treated with control and *GNG12-AS1* siRNAs (exons 1 and 7). The analysis was performed with six biological replicates for each cell line. cDNA synthesis, labelling and array procedure were conducted at the Genomic Facility at the CRUK CI. Pathway analysis was performed using Metacore.

### MeDIP (methylated DNA immunoprecipitation) qRT–PCR

Genomic DNA was extracted from cells with control and *GNG12-AS1* siRNA treated with 100 μg ml^−1^ RNaseA (Invitrogen) in 100 μl of Tris-EDTA for 10 min at room temperature and sonicated for 5 min (Diagenode Picoruptor, 30 s ON-30sec OFF) to obtain an average fragments size of 300 bp. DNA was purified using 1.8 volumes AMPURE XP beads (Beckman Coulter) according to the manufacturer's instructions and resuspended in 185 μl of water. The samples were boiled for 10 min and snap frozen on ice for 10 min. Five microlitres were taken as input followed by addition of 20 μl cold 10 × meDIP buffer. Meanwhile, 20 μl of Protein G Dynal beads (Invitrogen) were blocked with PBS/0.01% BSA for 30 min at room temperature and washed three times with 1 × meDIP buffer. One microlitre of anti-5mC antibody (Diagenode) was conjugated to the washed beads in 200 μl of 1 × meDIP buffer for 2 h at room temperature. Antibody-bead conjugates were washed three times with cold 1 × meDIP buffer, added to the sample DNA and incubated overnight at 4 °C in an overhead rotator. The bound material was washed three times with 200 μl of 1 × meDIP buffer at room temperature. Bound and input material were resuspended in 50 μl lysis solution (100 mM Tris-HCl, pH 5.5, 5 mM EDTA, 200 mM NaCl, 0.2% SDS plus 20 μg proteinase K (Invitrogen 25530-049)) and incubated at 60 °C for 30 min. DNA was purified using 1.8 volumes AMPURE XP beads (Beckman Coulter) according to the manufacturer's instructions. Validation of Medip was done by qRT*–*PCR. The list of Medip primers is present in Supplementary Table 2.

### Analysis of allelic expression and DNA methylation

Analysis of DNA methylation was by bisulfite conversion with the EZ DNA Methylation-Gold Kit (Zymo Research) and subsequent pyrosequencing as described^31^. The list of primers used for this experiment is present in Supplementary Table 5.

### RNA immunoprecipitation

HB2 cells were transfected with control and *GNG12-AS1* siRNAs and RIP was performed from nuclear extracts using mouse IgG (Cell Signaling) and AGO2 (Abcam) antibodies. Quality of cytoplasmic and nuclear extracts was assessed by immunoblot as described previously^65^. Eight micrograms of antibody were incubated with 70 μl of Dynabeads Protein G beads (Life Technologies) in total volume of 280 μl for 30 min at room temperature. The antibody–bead complex was incubated with 500 μg of nuclear exactas for 2–3 h at 4 °C and then washed three times (5 min each) with equal amount of 1 × NLB buffer (20 mM Tris-HCl, pH7.5, 0.15 M NaCl, 3 mM MgCl_2_, 0.3% NP-40, 10% glycerol) supplemented with Complete EDTA free protease inhibitor cocktail (14549800; Roche) and phosphatase inhibitors (1 mM NaF, 1 mM Na_3_VO_4_) and RNAse OUT (100 U; Life Technologies). The RNA was extracted by addition of 1 ml TRIzol (Life Technologies) to the beads, followed by 1/5 volume of Chloroform (Sigma). 10% of the input lysate was mixed with 1 ml of TRIzol for the total input RNA. After centrifugation at 12,000*g* for 15 min at 4 °C, the aqueous supernatant was transferred to a new tube and RNA was precipitated with 1/10 volume of 3 M sodium acetate pH 5.5 (Life Technologies), 1 volume of isopropanol (Sigma) and 1 μl of GlycoBLue (Life Technologies) at −80 °C for 20 min. After 30 min centrifugation at 12,000*g* at 4 °C, the RNA pellet was washed twice with ice-cold 70% ethanol. Finally, the pellet was dissolved in 15 μl of RNAse-free water (Life Technologies). We used Power SYBR Green RNA-to-CT 1-Step Kit (Life Technologies) for qRT–PCR. Primers for this assay were *GNG12-AS1* (exons 7–8) and *U1* and *GAPDH* (negative controls for AGO2 binding). Primers are listed in Supplementary Table 2.

### Cell lysis and immunoblot

Total cell lysis and immunoblot were performed as described previously^31^. Briefly, the cells were lysed in lysis buffer (50 mM Tris-HCl, pH 8, 125 mM NaCl, 1% NP-40, 2 mM EDTA, 1 mM phenylmethylsulphonyl fluoride, and protease inhibitor cocktail (Roche)) and incubated on ice for 25 min. The proteins were denatured, reduced and separated using Nupage Novex 4–12% Bis-Tris Protein Gels (Invitrogen). Secondary antibodies were conjugated with peroxidase, and immunobands were detected with a Supersignal West Dura HRP Detection Kit (Thermo-Scientific). Quantification of immunoblots was done on ImageScanner III (GE HealthCare) using the software package ImageQuant TL 7.0 (GE HealthCare). Uncropped scans of the immunoblots are shown in Supplementary Fig. 12.

### Antibodies

The list of antibodies is present in Supplementary Table 6.

### Chromatin immunoprecipitation

ChIP assays were performed as previously described^31^ with antibodies listed in the Supplementary Table 6. The input and the immunoprecipitated materials were quantified by QubitFluorometer (Life Technologies) with the dsDNA BR Assay Kit (Invitrogen). Thirty micrograms of chromatin and 5 μg of antibody were used for ChIP experiment. The qPCR data were corrected for DNA amount, and enrichment was normalized against the input according to the formula 2-dCt(Ab)—log2(DF)]—[Ct(input)—log2(DF)]. Primer sequences are present in Supplementary Table 2.

### Cell synchronization

The HB2 cells were plated in six-well plates and 24 h later thymidine (2 mM) was added. The cells were incubated for 18 h, washed three times with PBS and released into thymidine-free medium for 9 h. Thymidine was then added for a further 15 h. The cells were then washed three times with PBS. At this point, the cells in G1/S phase were collected for RNA, FACS and protein analysis (T0) in a serum-rich medium without thymidine. Time points were collected 3 (T3), 8 (T8), 14 (T14), 24 (T24) and 32 (T32) hours later. In the case of serum starvation, HB2 cells were plated in six-well plates and then switched to serum-free medium for 48 h. After starvation (T0), the cells were released into cell cycle by addition of serum and the time points for RNA and protein analysis were collected at 3 (T3), 6 (T6), 10 (T10), 15 (T15) and 34 (T34) hours. Cyclin E1 (G1/S transition marker) levels were used to monitor cell cycle progression by immunoblot. In the case of siRNA treatment, the cells were first transfected with siRNAs and thymidine was added 24 h later for additional 18 h. The procedure was continued as described above.

### Cell cycle analysis

The HB2 cells were transfected with siRNAs and harvested after 72 h, washed with PBS and fixed with 70% ethanol (the samples were stored for up to 1 week at 4 °C). The cells were then washed once with PBS, incubated in PBS containing RNase A (100 μg ml^−1^, Life Tecnologies) for 30 min at 37 °C, stained with propidium iodide (20 μg ml^−1^, Life Tecnologies) and incubated on ice in the dark for 30 min. DNA content was analysed by FACS Calibur (Beckton Dickinson) using BD CellQuest Pro Software V6. DNA cell cycle analysis was performed on FlowJo software V9 (TreeStar Inc) to quantify cell cycle distribution.

### Wound healing assay

Wound healing assay was performed on 24-well (Essen Imagelock) plates in triplicates after treatment of cells with control and siRNAs against *GNG12-AS1*. Forty-eight hours after siRNA treatment, scratch wounds were induced with 10 μl sterile pipette tip, after which fresh culture medium was added. The IncuCyte 2011A Rev2 software was used to capture and analyse the pictures. The cell migration was followed in time for 24 h using IncuCyte FLR (Essen Bioscience), making measurements in triplicate every 3 h.

### Statistical analysis

The statistical significance of data was determined by two-tailed Student's *t*-test in all experiments using GraphPad Prism unless indicated otherwise. *P*-values>0.05 were considered statistically not significant.

## Additional information

**Accession codes:** The microarray data have been deposited in the Gene Expression Omnibus of National Center for Biotechnology Information under the accession number GSE60563.

**How to cite this article:** Stojic, L. *et al.* Transcriptional silencing of long noncoding RNA *GNG12-AS1* uncouples its transcriptional and product-related functions. *Nat. Commun.* 7:10406 doi: 10.1038/ncomms10406 (2016).

## Supplementary Material

Supplementary Information Supplementary Figures 1-12 and Supplementary Tables 1-6

Supplementary Data 1 Sequences of the GNG12-AS1 splice variants and scrambled vector
