# Supplementary material for: Transcriptional silencing of long noncoding RNA GNG12-AS1 uncouples its transcriptional and product-related functions
Source: Nat Commun. 2016 Feb 2;7:10406. doi: 10.1038/ncomms10406 (PMC4740813; doi:10.1038/ncomms10406)
Supplement: Supplementary Information — Supplementary Figures 1-12 and Supplementary Tables 1-6 [file ncomms10406-s1.pdf]

Supplementary Figures

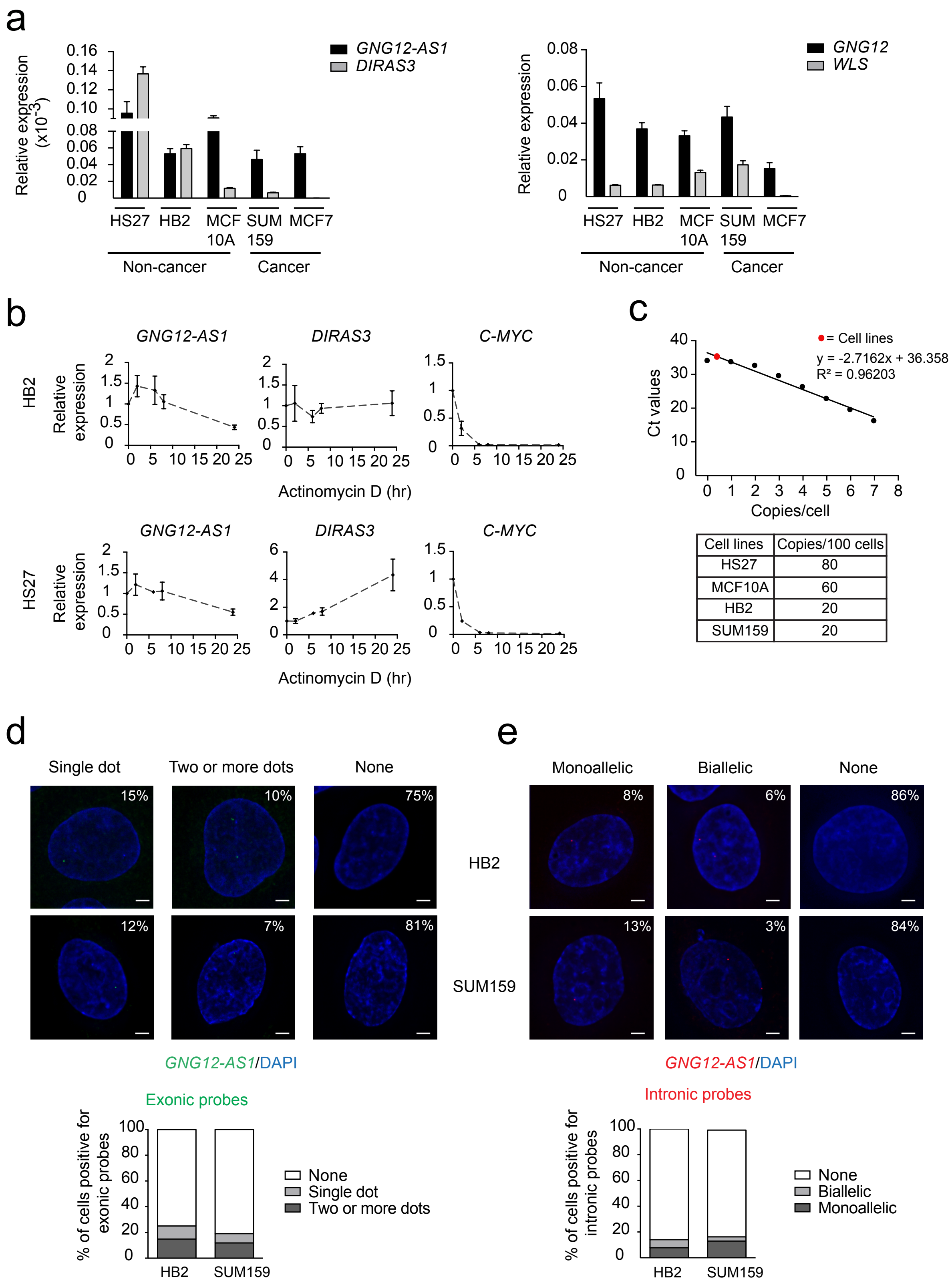

Cell lines	Copies/100 cells
HS27	80
MCF10A	60
HB2	20
SUM159	20

Supplementary Figure 1. *GNG12-AS1* is a stable nuclear lncRNA

a) Expression levels of *DIRAS3*, *GNG12-AS1, GNG12 and WLS* analysed by qRT-PCR in non-cancer (HS27, HB2, MCF10A) and cancer cell lines (SUM159, MCF7) and normalised to the geometric mean of *GAPDH and RPS18.* Primers located in exon 7-8 were used for *GNG12-AS1* expression. Error bars, s.e.m. (n = 3 biological replicates).

b) Stability of *GNG12-AS1* as determined after treatment of HB2 (top) and HS27 (bottom) cells with Actinomycin D (10µg/ml). The half-life of *GNG12-AS1* is between 20 and 25 hr. *C-MYC* has a short half-life and is a positive control for Actinomycin D treatment. Expression levels were measured by qRT-PCR, normalised to *GAPDH* and standardised to time point 0 hr (set as 1). Error bars, s.e.m. (n = 3 biological replicates).

c) Calculation of *GNG12-AS1* copy number using a standard curve method of plotting Ct values vs dilutions of a cloned *GNG12-AS1* DNA template of known concentration. *GNG12-AS1* expression per cell was calculated from two independent qRT-PCR experiments using RNA extracted from known number of cells. *GNG12-AS1* is present at 20 (HB2, SUM159), 60 (MCF10A) or 80 (HS27) molecules per 100 cells. (n = 2 biological replicates).

d, e) Single-molecule RNA FISH of *GNG12-AS1* in the normal breast epithelial cell line HB2 and breast cancer cell line SUM159 using exon-specific probes (green, **d**) and intronic probes (red, **e**). The numbers represent the percentage of cells with positive FISH signals (n = 1959 cells for HB2; n = 895 cells for SUM159). The rest of the cells show no signal (75-81% for exonic probes; 84-86% for intronic probes). The nucleus was stained with DAPI. Scale bar, 3.3µm.

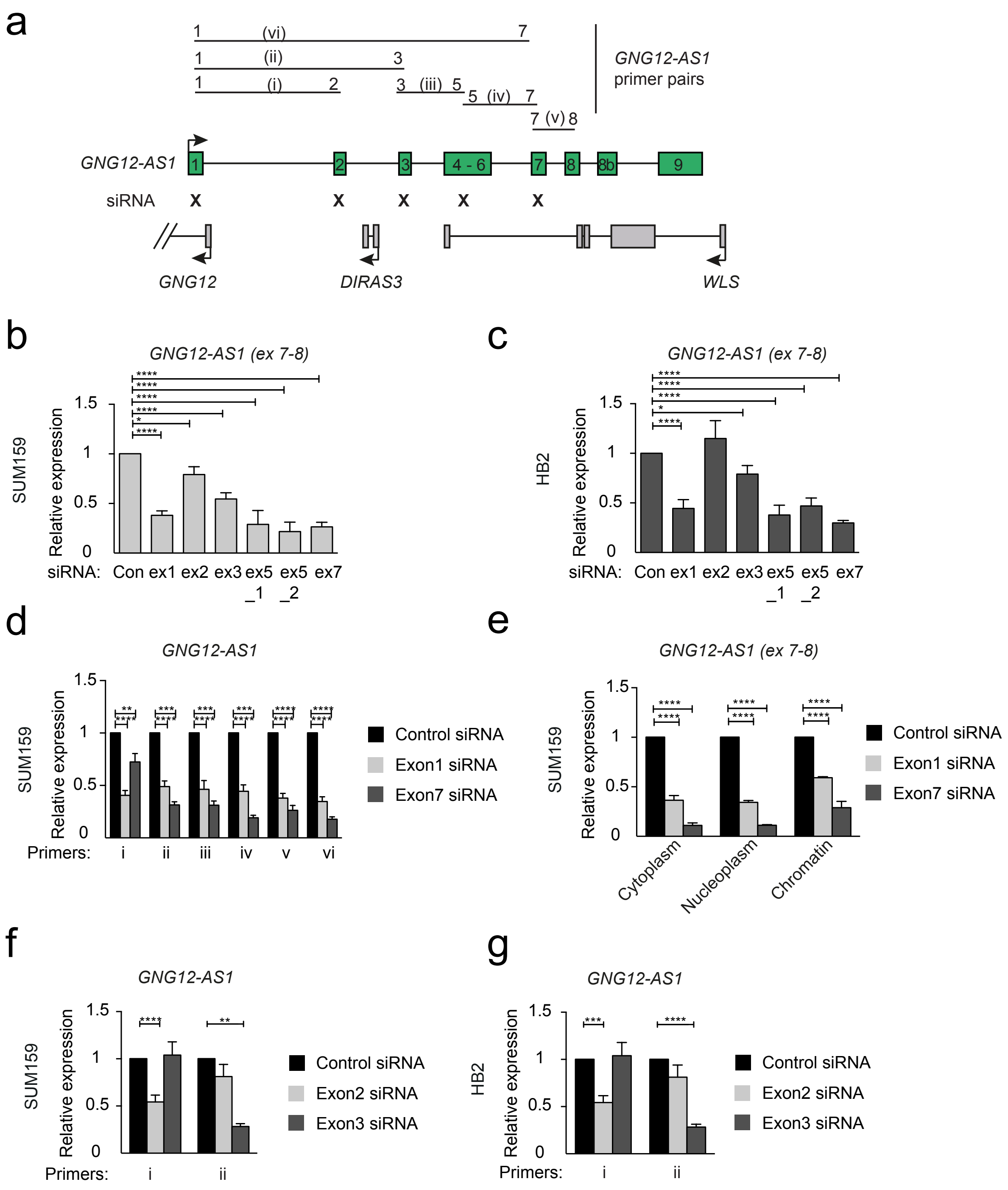
a
GNG12-AS1 primer pairs
GNG12-AS1
siRNA
GNG12
DIRAS3
WLS
b
GNG12-AS1 (ex 7-8)
SUM159
Relative expression
siRNA: Con ex1 ex2 ex3 ex5_1 ex5_2 ex7
c
GNG12-AS1 (ex 7-8)
HB2
Relative expression
siRNA: Con ex1 ex2 ex3 ex5_1 ex5_2 ex7
d
GNG12-AS1
SUM159
Relative expression
Primers: i ii iii iv v vi
Control siRNA
Exon1 siRNA
Exon7 siRNA
e
GNG12-AS1 (ex 7-8)
SUM159
Relative expression
Cytoplasm
Nucleoplasm
Chromatin
Control siRNA
Exon1 siRNA
Exon7 siRNA
f
GNG12-AS1
SUM159
Relative expression
Primers: i ii
Control siRNA
Exon2 siRNA
Exon3 siRNA
g
GNG12-AS1
HB2
Relative expression
Primers: i ii
Control siRNA
Exon2 siRNA
Exon3 siRNA

Supplementary Figure 2. Validation of *GNG12-AS1* knockdown by siRNAs targeting different exons of *GNG12-AS1*

a) Schematic representation of the *GNG12-AS1* genomic locus relative to *GNG12, DIRAS3* and *WLS* together with siRNAs targeting different exons of *GNG12-AS1* and primers used for *GNG12-AS1* expression.

b, c) siRNA-mediated knockdown of exons 1, 2, 3, 5 and 7 of *GNG12-AS1* in SUM159 (**b**) and HB2 (**c**). Primers spanning exon 7-8 were used for *GNG12-AS1* expression.

d) siRNA-mediated knockdown of exon 1 and exon 7 of *GNG12-AS1* in SUM159. Primers spanning exon 1-2 (i), 1-3 (ii), 3-5 (iii), 5-7 (iv), 7-8 (v) and 1-7(vi) were used to capture several isoforms of *GNG12-AS1*. The majority of *GNG12-AS1* isoforms are depleted with both siRNAs. *GNG12-AS1* isoforms containing exon 2 are affected to a lesser extent by siRNA targeting exon 7.

e) Quantification of *GNG12-AS1* depletion by siRNA targeting exon 1 or exon 7 by qRT-PCR in subcellular fractions. Relative RNA levels are standardised to the geometric mean of *GAPDH* and *β-actin*. Primers spanning exon 7-8 were used for *GNG12-AS1* expression. Error bars, s.e.m. (n = 4 biological replicates).

f, g) siRNA targeting exon 2 and exon 3 of *GNG12-AS1* in SUM159 (**f**) and HB2 (**g**). Primers spanning exon 1-2 (i) and 1-3 (ii) were used for *GNG12-AS1* expression.

For all the graphs (except **e**), the expression of *GNG12-AS1* was normalised to *GAPDH* and compared to control siRNA by qRT-PCR. The statistical significance of the individual bars is compared to their respective control bars. Error bars, s.e.m. (n = 3 biological replicates). $^{*}p < 0.05$, $^{**}p < 0.01$, $^{***}p < 0.001$ and $^{****}p < 0.0001$ by two-tailed Student's t-test.

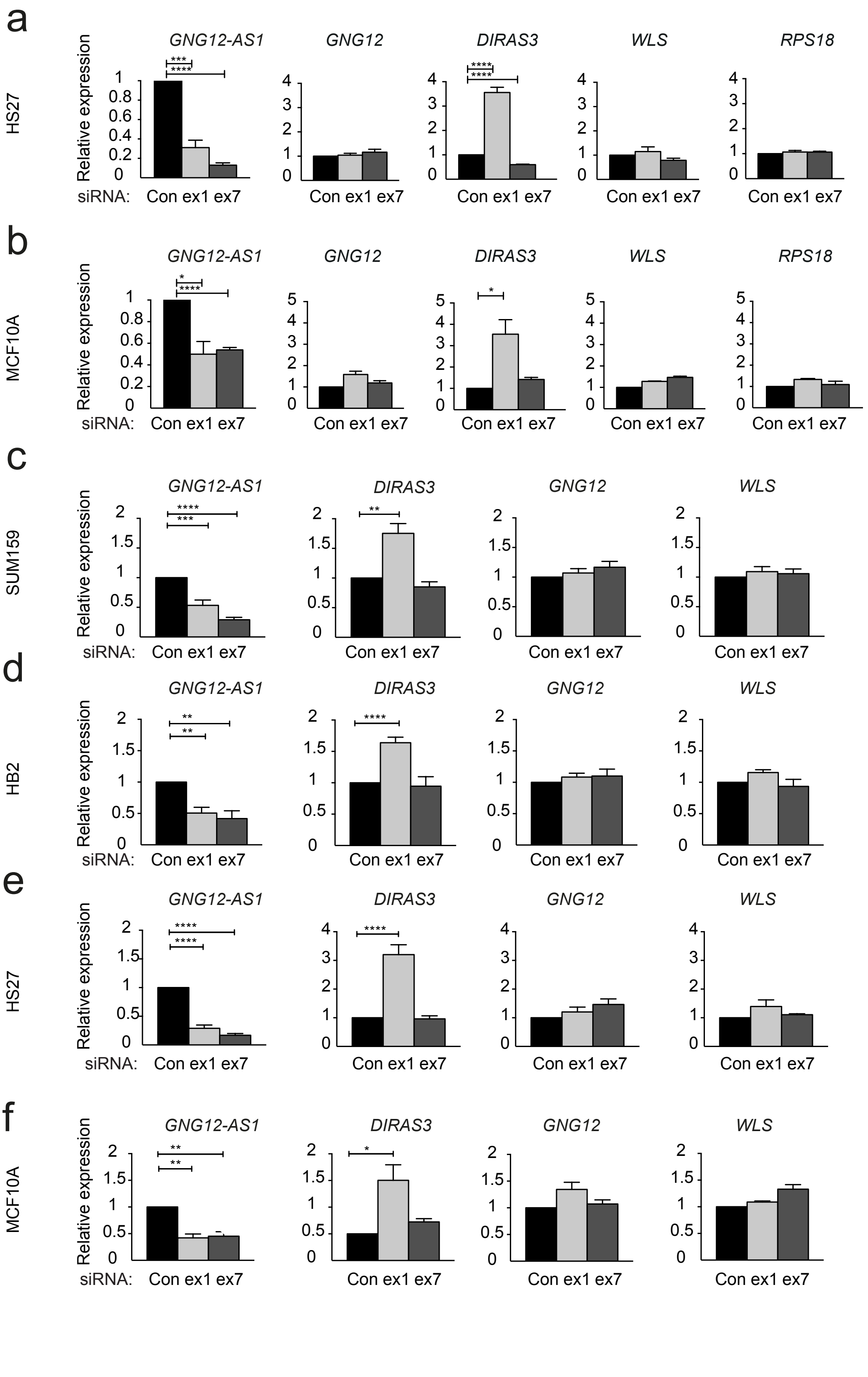
a
HS27
Relative expression
GNG12-AS1
GNG12
DIRAS3
WLS
RPS18
siRNA:
Con ex1 ex7
b
MCF10A
c
SUM159
d
HB2
e
HS27
f
MCF10A

Supplementary Figure 3. Depletion of *GNG12-AS1* by siRNAs targeting exon 1 and exon 7 in different cell lines

a-b) siRNA targeting exon 1 and exon 7 of *GNG12-AS1* in HS27 (**a**) and MCF10A (**b**). Expression levels of *DIRAS3, GNG12* and *WLS* were normalised to *GAPDH* and compared to control siRNA by qRT-PCR.

c-f) siRNA targeting exon 1 and exon 7 of *GNG12-AS1* in SUM159 (**c**), HB2 (**d**), HS27 (**e**) and MCF10A (**f**). Expression levels of *DIRAS3, GNG12* and *WLS* were normalised to the geometric mean of *GAPDH* and *RPS18* and compared to control siRNA by qRT-PCR. *DIRAS3* was upregulated only with siRNA targeting exon 1, but not exon 7, of *GNG12-AS1* regardless if one (*GAPDH)* or two reference genes (*GAPDH* and *RPS18*) were used for normalisation.

For all the graphs (**a-f**), the statistical significance of the individual bars is compared to their respective control bars. Error bars, s.e.m. (n = 3 biological replicates). $^*p < 0.05$, $^{**}p < 0.01$, $^{***}p < 0.001$ and $^{****}p < 0.0001$ by two-tailed Student's t-test.

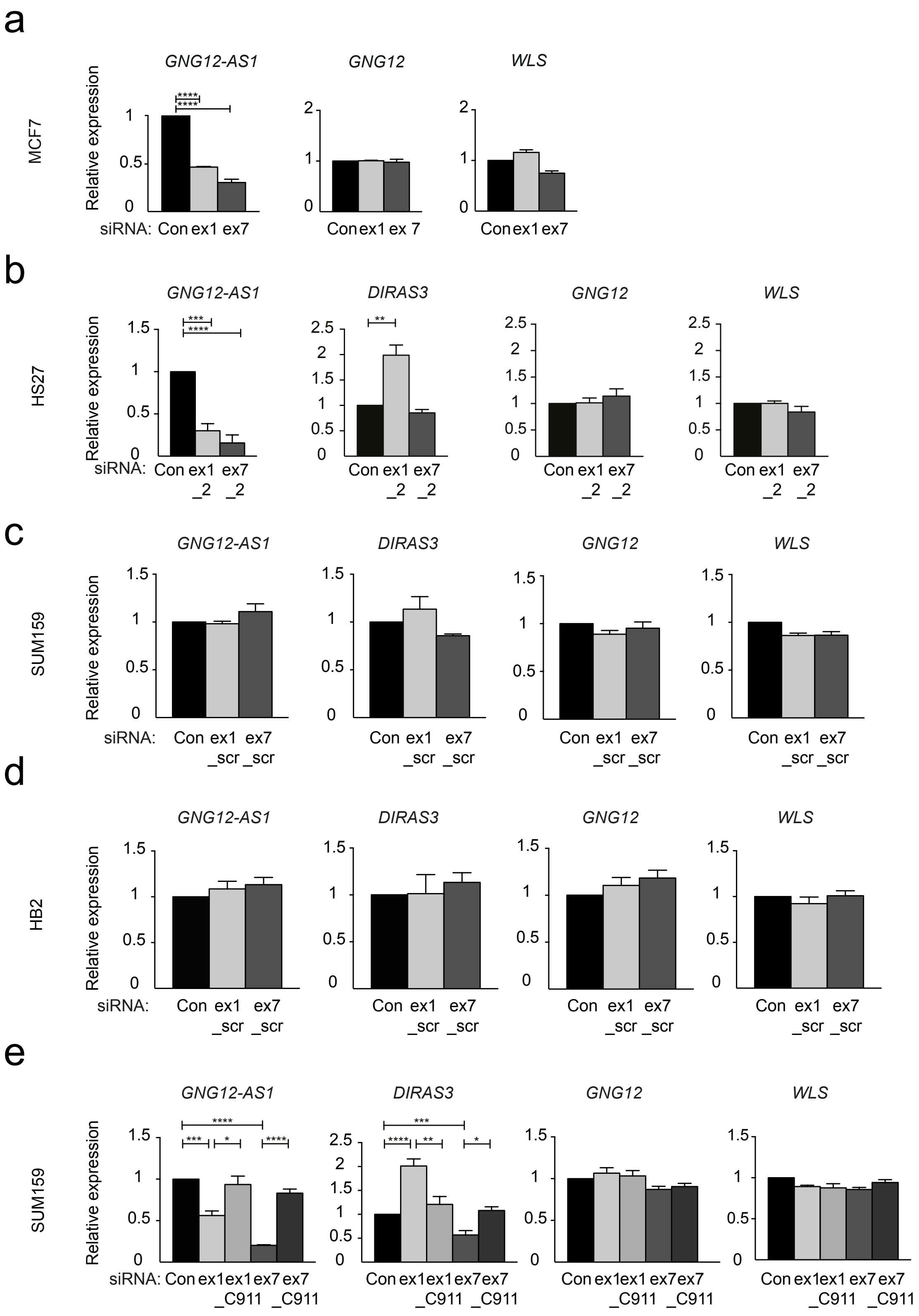
a
MCF7
GNG12-AS1
GNG12
WLS
Relative expression
siRNA: Con ex1 ex7
Con ex1 ex 7
Con ex1 ex7
b
HS27
GNG12-AS1
DIRAS3
GNG12
WLS
Relative expression
siRNA: Con ex1_2 ex7_2
c
SUM159
GNG12-AS1
DIRAS3
GNG12
WLS
Relative expression
siRNA: Con ex1_scr ex7_scr
d
HB2
GNG12-AS1
DIRAS3
GNG12
WLS
Relative expression
siRNA: Con ex1_scr ex7_scr
e
SUM159
GNG12-AS1
DIRAS3
GNG12
WLS
Relative expression
siRNA: Con ex1 ex1_C911 ex7 ex7_C911

Supplementary Figure 4. Additional siRNA controls to exclude off-target effects after *GNG12-AS1* depletion

a) siRNA targeting exon 1 and exon 7 of *GNG12-AS1* in MCF7 which is hypermethylated for *DIRAS3*. Depletion of *GNG12-AS1* in MCF7 cells did not reactivate *DIRAS3.*

b) Additional siRNAs against exon 1 (ex1_2) and exon 7 (ex7_2) of *GNG12-AS1* exclude off-target effects and confirm that *DIRAS3* expression is upregulated when exon 1 is targeted.

c, d) Off-target effects were excluded by treating the cells with randomised nucleotide sequence (scrambled siRNAs of exons 1, ex1_scr and exon 7, ex7_scr) in SUM159 (**c**) and HB2 cells (**d**) cells.

e) SUM19 cells were treated with exon 1 and exon 7 siRNAs and their corresponding "C911" controls which have the same siRNA seed region as exon 1 and exon 7 except bases 9 to 11 are replaced with their complement bases. The depletion of *GNG12-AS1* and *DIRAS3* expression was not affected by "C911" version of exon 1 and exon 7 siRNAs excluding *DIRAS3* upregulation as an off-target of *GNG12-AS1* targeting by siRNAs.

For all the graphs (**a-e**), the expression levels of *GNG12-AS1, GNG12* and *WLS* were normalised to *GAPDH* and compared to control siRNA by qRT-PCR. Primers spanning exon 7-8 were used for *GNG12-AS1* expression. The statistical significance of the individual bars is compared to their respective control bars. Error bars, s.e.m. ($n = 3$ biological replicates). $^*p < 0.05$, $^{**}p < 0.01$, $^{***}p < 0.001$ and $^{****}p < 0.0001$ by two-tailed Student's t-test.

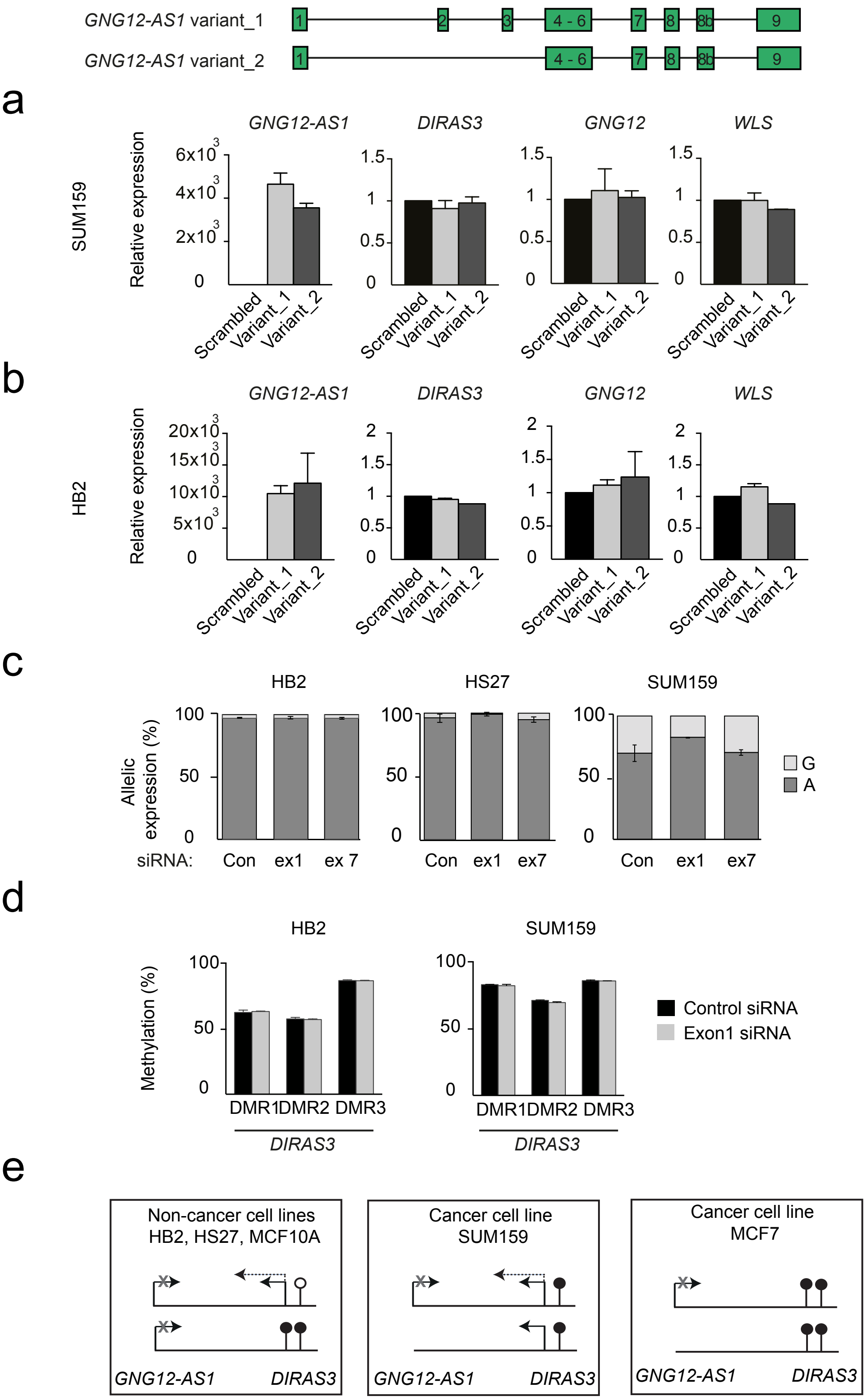

GNG12-AS1 variant_1
GNG12-AS1 variant_2
a
SUM159
Relative expression
GNG12-AS1
DIRAS3
GNG12
WLS
Scrambled
Variant_1
Variant_2
b
HB2
c
HB2
HS27
SUM159
Allelic expression (%)
siRNA:
Con
ex1
ex 7
ex7
G
A
d
Methylation (%)
DMR1
DMR2
DMR3
DIRAS3
Control siRNA
Exon1 siRNA
e
Non-cancer cell lines HB2, HS27, MCF10A
Cancer cell line SUM159
Cancer cell line MCF7

Supplementary Figure 5. *GNG12-AS1* regulates *DIRAS3* expression in *cis*

a, b) Exogenous overexpression of spliced forms of *GNG12-AS1* does not affect *DIRAS3* expression. *GNG12-AS1, DIRAS3, GNG12* and *WLS* expression was analysed with qRT-PCR after viral overexpression of two different isoforms of *GNG12-AS1* in SUM159 (**a**) and HB2 cells (**b**). The expression was normalised to scrambled vector (negative control) after standardisation to *GAPDH*. *GNG12-AS1* variant _1 includes exons 1, 2, 3, 4, 5, 5b, 6, 7, 8, 8b, 9, whereas variant _2 which is the most abundant splice isoform, includes exons 1, 4, 5, 5b, 6, 7, 8, 8b, 9. Error bars, s.e.m. (n = 3 biological replicates for SUM159; n = 2 biological replicates for HB2).

c) *DIRAS3* imprinting does not change after siRNA targeting 5' end of *GNG12-AS1,* indicating that the active allele is modulated by *GNG12-AS1* in *cis.* Allelic expression of *DIRAS3* upon *GNG12-AS1* knockdown was measured by pyrosequencing (PSQ) in cell lines with informative SNP (rs11801053): HS27, HB2 and SUM159. The imprinted silent allele is not reactivated. In SUM159 cells increased *DIRAS3* expression was associated with a slight skew to monoallelic expression of *DIRAS3* after exon 1 siRNA treatment (control siRNA = 70:30%; exon 1 siRNA = 82:17%). Error bars, s.e.m. (n = 3 biological replicates).

d) *DIRAS3* methylation does not change after siRNA targeting 5' end of *GNG12-AS1.* Methylation of differentially methylated regions (DMRs) DMR1, DMR2 and DMR3 of *DIRAS3* does not change after depletion of *GNG12-AS1*. Methylation levels were analysed by PSQ of bisulfite converted genomic DNA in HB2 and SUM159 cells after control and exon 1 *GNG12-AS1* siRNA treatment. Error bars, s.e.m. (n = 3 biological replicates).

e) Summary of how siRNA directed to exon 1 of *GNG12-AS1* modulates the expression of the active *DIRAS3* allele in *cis*, rather that reactivating the silent allele. Each panel shows the methylation and expression states of the two parental alleles in different cell lines. The arrows indicate expression and lollipops show methylation (filled) and no methylation (unfilled). A single lollipop is partial methylation, a double lollipop is full methylation. The x above the *GNG12-AS1* arrows indicates siRNA inhibition of exon 1 and the dotted arrows above *DIRAS3* depicts increased transcription after this inhibition.

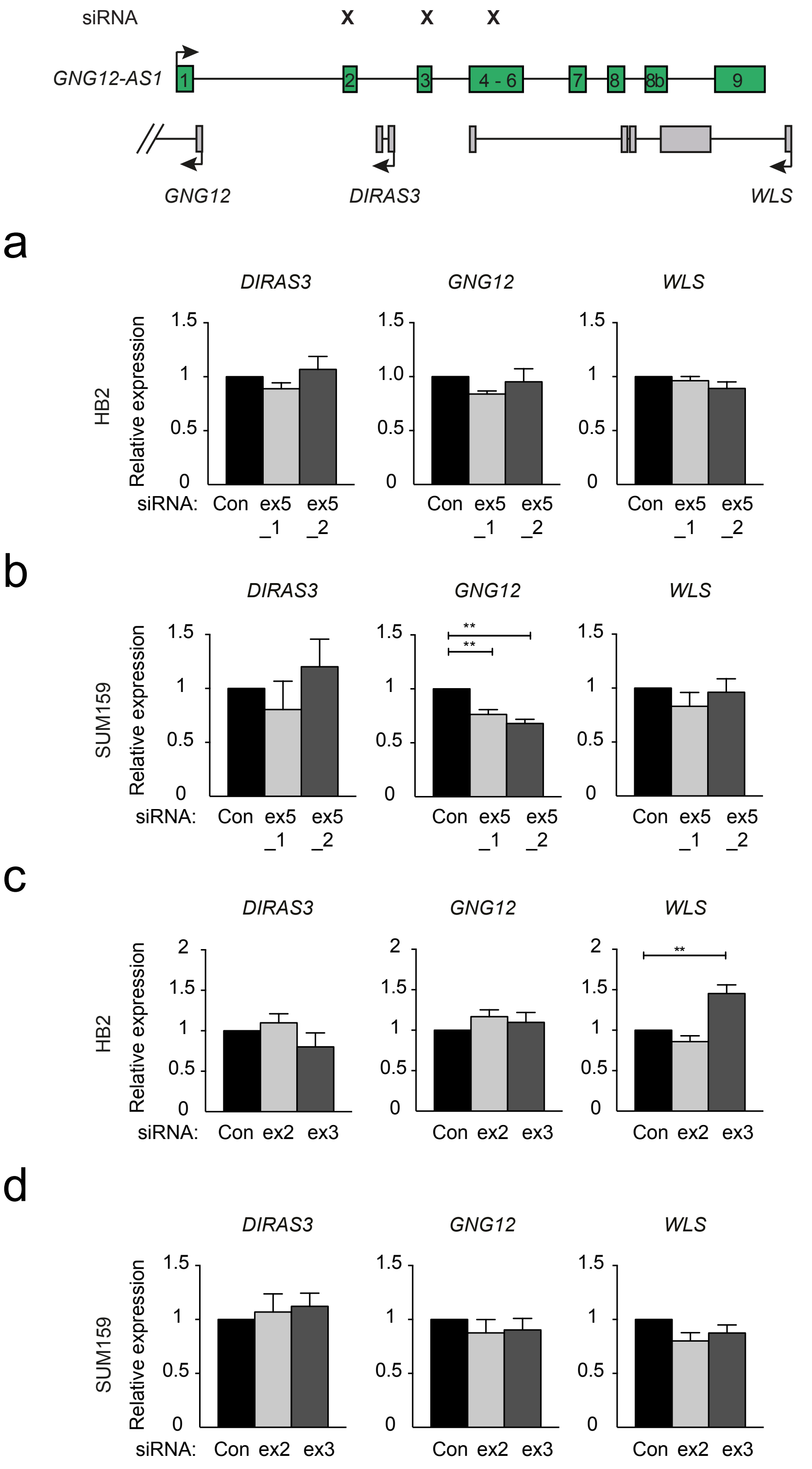
siRNA
x x x
GNG12-AS1
1 2 3 4 - 6 7 8 8b 9
GNG12
DIRAS3
WLS
a
b
c
d
HB2
SUM159
Relative expression
DIRAS3
GNG12
WLS
siRNA:
Con ex5_1 ex5_2
Con ex2 ex3
**

Supplementary Figure 6. Targeting exons 2, 3 or 5 of *GNG12-AS1* does not affect *DIRAS3* expression

a, b) HB2 (**a**) and SUM159 (**b**) cells were transfected with two different siRNA targeting exon 5 of *GNG12-AS1*. No effect on *DIRAS3* expression was observed but *GNG12* was modestly reduced in SUM159 cells. The knockdown efficiency of siRNAs to exon 5 is shown in Supplementary Fig. 2b and c.

c, d) HB2 (**c**) and SUM159 (**d**) cells were treated with siRNAs targeting exon 2 and exon 3 of *GNG12-AS1.* Primers spanning exon 1-2 and exon 1-3 were used for *GNG12-AS1* expression. The knockdown efficiency of siRNAs to exons 2 and 3 is shown in Supplementary Fig. 2f. siRNA to exon 3 of *GNG12-AS1* in HB2 cells modestly upregulated *WLS.* It is likely that specific *GNG12-AS1* isoforms can cell type specifically affect the expression of neighboring genes. We have not validated cell specific effects in this study.

For all the graphs (**a-d**), expression of *DIRAS3*, *GNG12* and *WLS* was normalised to *GAPDH* and compared to control siRNA by qRT-PCR. The statistical significance of the individual bars is compared to their respective control bars. Error bars, s.e.m. ($n = 3$ biological replicates). $^{**}p < 0.01$ by two-tailed Student's t-test.

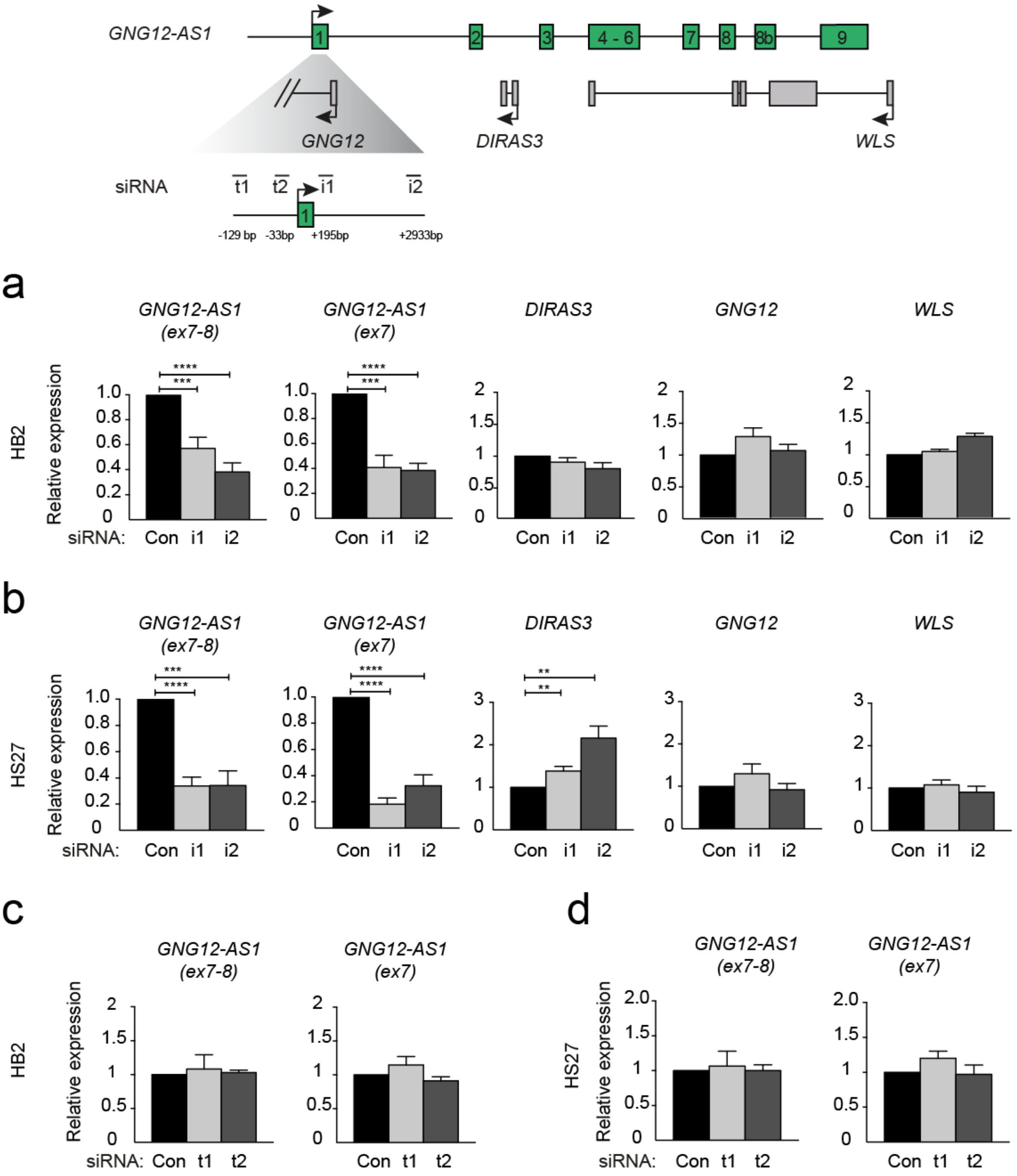
GNG12-AS1
1
2
3
4 - 6
7
8
8b
9
GNG12
DIRAS3
WLS
siRNA
t1
t2
i1
i2
-129 bp
-33bp
+195bp
+2933bp
a
b
c
d
GNG12-AS1 (ex7-8)
GNG12-AS1 (ex7)
DIRAS3
GNG12
WLS
HB2
HS27
Relative expression
siRNA:
Con
i1
i2
t1
t2

Supplementary Figure 7. Differential effect of siRNAs targeting 5' or 3' end of exon 1 of *GNG12- AS1*

a, b) The depletion of nascent *GNG12-AS1* transcript in HB2 (**a**) and HS27 (**b**) cells was achieved by siRNAs targeting intronic region 195 bp (i1) and 2933 bp (i2) downstream of *GNG12-AS1* TSS. *DIRAS3* expression changes occured only in HS27 cells.

c, d) Targeting promoter region in HB2 (**c**) and HS27 (**d**) cells with siRNAs located 33 bp (t2) and 129 bp (t1) upstream of *GNG12-AS1* TSS had no effect on *GNG12-AS1* expression.

For all the graphs (**a-d**), expression of *DIRAS3, GNG12-AS1*, *GNG12* and *WLS* were normalised to *GAPDH* and compared to control siRNA by qRT-PCR. Primers spanning exon 7-8 or primers against exon 7 were used for *GNG12-AS1* knockdown efficiency. The statistical significance of the individual bars is compared to their respective control bars. Error bars, s.e.m. (n = 3 biological replicates). **$p < 0.01$, ***$p < 0.001$ and ****$p < 0.0001$ by two-tailed Student's t-test.

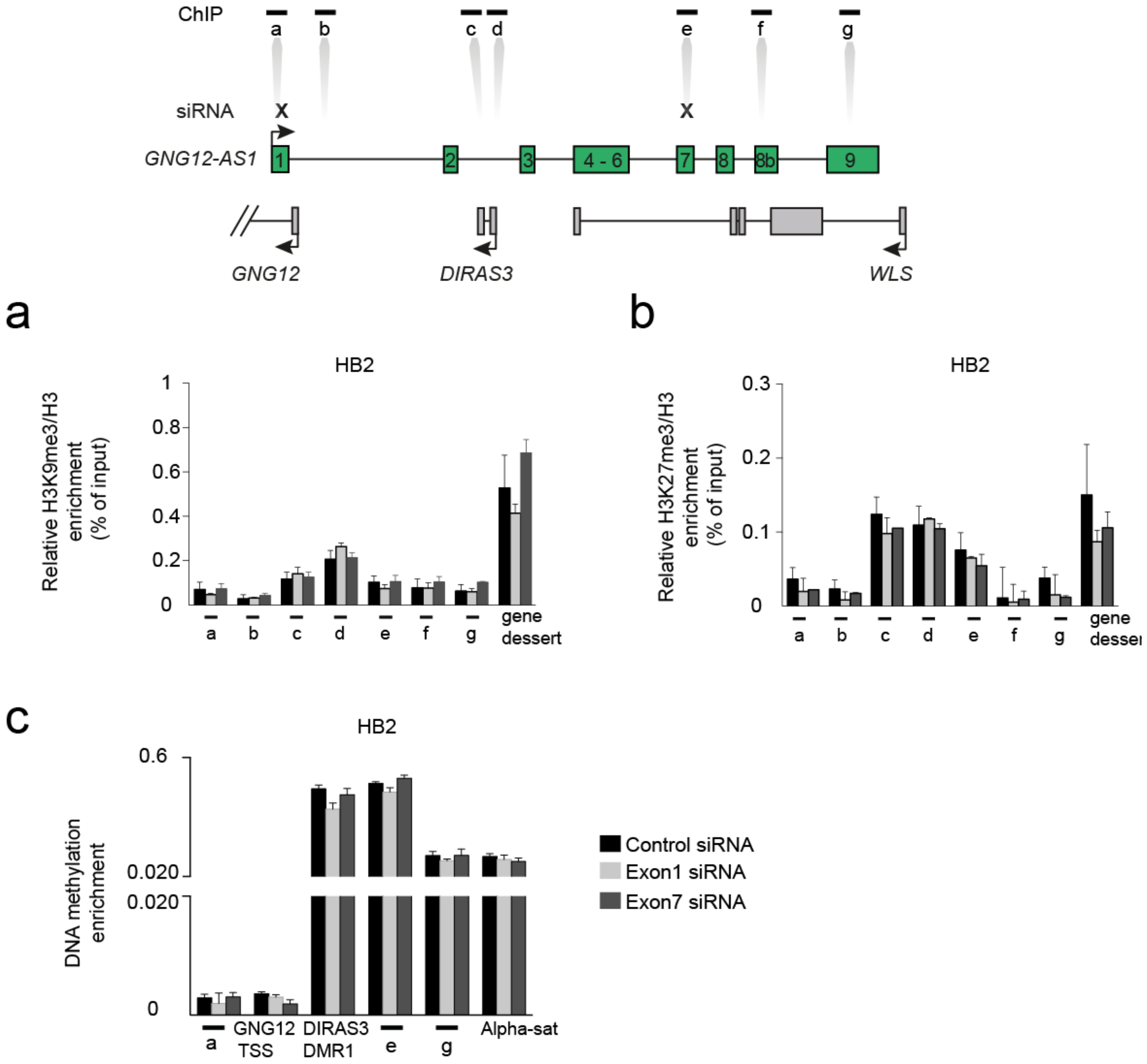
ChIP
a b c d e f g
siRNA
X X
GNG12-AS1
1 2 3 4 - 6 7 8 8b 9
GNG12
DIRAS3
WLS
a
HB2
Relative H3K9me3/H3 enrichment (% of input)
1 0.8 0.6 0.4 0.2 0
a b c d e f g gene dessert
b
HB2
Relative H3K27me3/H3 enrichment (% of input)
0.3 0.2 0.1 0
a b c d e f g gene dessert
c
HB2
DNA methylation enrichment
0.6 0.020 0.020 0
a GNG12 TSS DIRAS3 DMR1 e g Alpha-sat
Control siRNA
Exon1 siRNA
Exon7 siRNA

Supplementary Figure 8. Analysis of repressive histone modifications and DNA methylation after *GNG12-AS1* siRNA treatment

a, b) H3K9me3 and H3K27me3 levels do not change after *GNG12-AS1* depletion. ChIP analysis was performed on chromatin prepared from control and exon 1 or exon 7 siRNA treated HB2 cells using H3K9me3 (**a**) and H3K27me3 (**b**) antibodies. The precipitated DNA fragments were subjected to qRT-PCR analysis with primers amplifying the *GNG12-AS1* TSS (a), region 1.6kb downstream of *GNG12-AS1* TSS (b), exon 2 of *DIRAS3* (c), *DIRAS3* TSS (d), *GNG12-AS1* exon 7 (e), *GNG12-AS1* exon 8b (f) and *GNG12-AS1* exon 9 (g). Levels of H3K9me3 and H3K27me3 were normalised to histone H3 density. Gene dessert was used as a positive control for repressive histone marks. ChIP enrichments are presented as the percentage of protein bound, normalised to input. Error bars, s.e.m. (n = 2 biological replicates).

c) Depletion of *GNG12-AS1* does not affect DNA methylation levels along *GNG12-AS1* locus. MedIP was performed on genomic DNA prepared from control and exon 1 or exon 7 siRNA treated HB2 cells. The precipitated methylated DNA fragments were subjected to qRT-PCR analysis with primers amplifying the *GNG12-AS1* TSS (a), *GNG12* TSS, *DIRAS3* DMR1, *GNG12-AS1* exon 7 (e) and *GNG12-AS1* exon 9 (g). MedIP enrichments are presented as the percentage of 5mC bound, normalised to input. α-satellite region was used as a positive control region for enrichment in DNA methylation. Error bars, s.e.m. (n = 2 biological replicates).

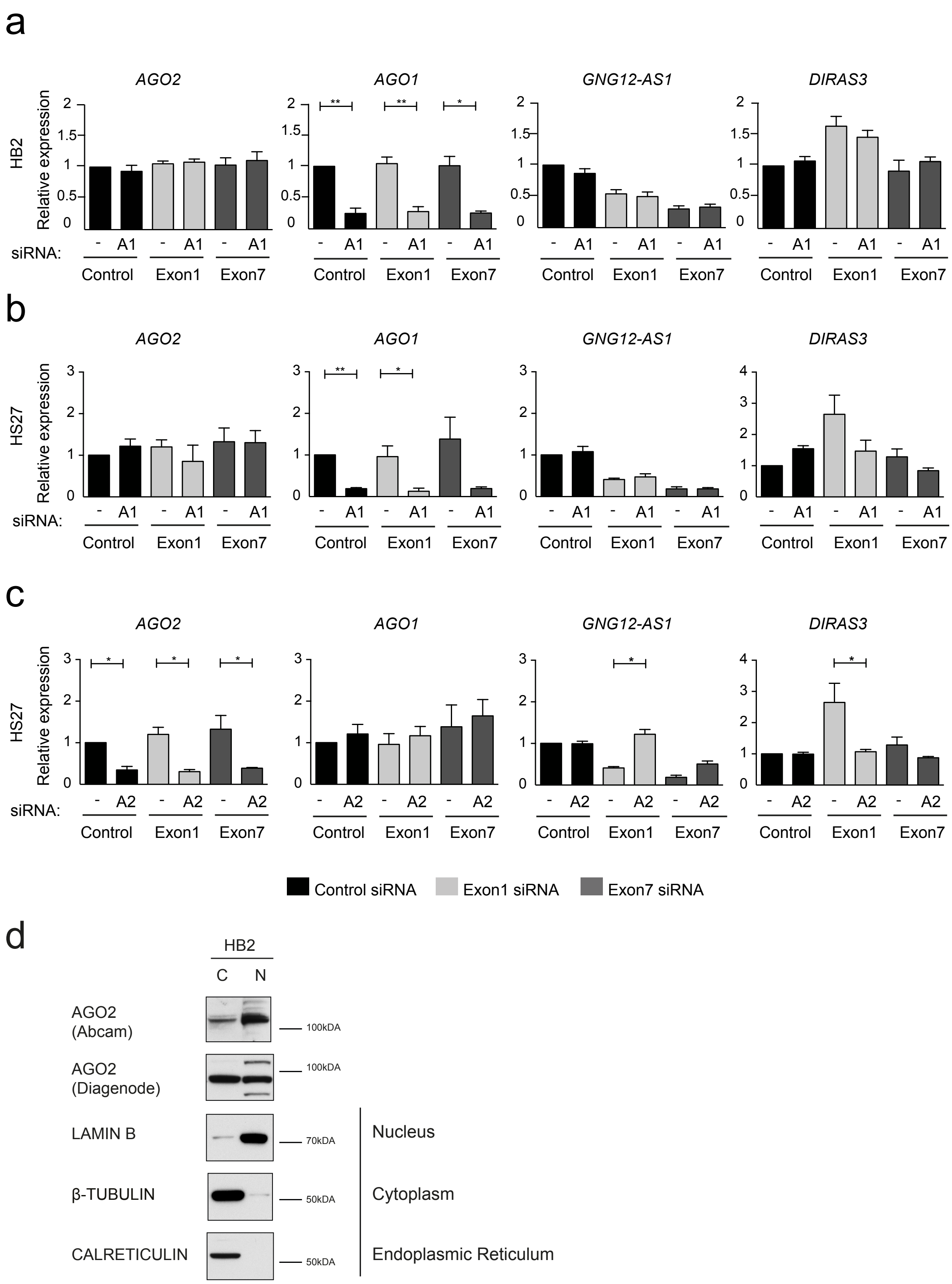
a
AGO2
AGO1
GNG12-AS1
DIRAS3
HB2
Relative expression
siRNA:
- A1
Control
Exon1
Exon7
b
HS27
c
A2
Control siRNA
Exon1 siRNA
Exon7 siRNA
d
HB2
C N
AGO2 (Abcam)
AGO2 (Diagenode)
LAMIN B
β-TUBULIN
CALRETICULIN
100kDA
70kDA
50kDA
Nucleus
Cytoplasm
Endoplasmic Reticulum

Supplementary Figure 9. Analysis of Argonaute proteins in siRNA-induced silencing of *GNG12-AS1*

a) HB2 cells were transfected with siRNA to exon 1 or exon 7 of *GNG12-AS1* together with control siRNA and siRNA targeting *AGO1 (A1)*. *AGO1* levels were not affected by either single *AGO2* siRNA or double knockdown of *AGO2* and *GNG12-AS1*. The double knockdown between *AGO1* and *GNG12-AS1* with siRNA targeting exon 1 did not rescue *DIRAS3* expression.

b, c) HS27 cells were transfected with exon 1 or exon 7 *GNG12-AS1* siRNA along with control siRNA and siRNA targeting *AGO1* (**b**) or *AGO2* (**c**). Single knockdown of *AGO1* or *AGO2* does not affect *GNG12-AS1* or *DIRAS3* expression while double knockdown of *GNG12-AS1* and *AGO2* can rescue *DIRAS3* expression.

d) Immunoblot analysis of AGO2 and subcellular markers prepared from cytoplasmic and nuclear fraction of HB2 cells. Two AGO2 antibodies (Abcam, Diagenode) were used, both confirming AGO2 presence in the nucleus. β-Tubulin is a marker of cytoplasm, lamin B of nucleus and calreticulin is a marker of endoplasmatic reticulum. C = cytoplasm; N = nucleus.

For all the graphs (**a-c**), expression levels of *AGO2, AGO1, DIRAS3* and *GNG12-AS1* were normalised to *GAPDH* and compared to control siRNA by qRT-PCR. Primers spanning exon 7-8 were used for *GNG12-AS1* expression. The statistical significance of the individual bars is compared to their respective control bars. Error bars, s.e.m. (n = 3 biological replicates). $^{*}p < 0.05$ and $^{**}p<0.01$ by two-tailed Student’s t-test.

a

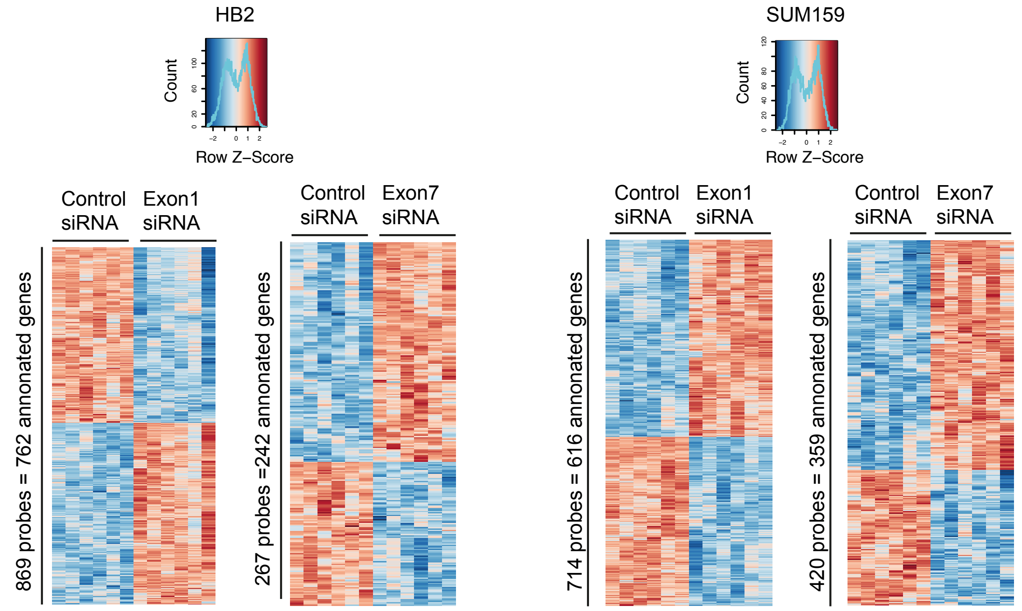

b

HB2 exon1 siRNA

	p value
Development_EMT_Regulation of epithelial-to-mesenchymal transition	2.781E-03
Development_Regulation of angiogenesis	4.387E-03
Inflammation_MIF signaling	6.066E-03
Cell adhesion_Integrin-mediated cell-matrix adhesion	1.078E-02
Cytoskeleton_Intermediate filaments	2.187E-02
Protein folding_Protein folding nucleus	2.750E-02
Signal transduction_Leptin signaling	3.300E-02
Translation_Translation in mitochondria	3.312E-02
Cell adhesion_Cell-matrix interactions	3.565E-02
Cell cycle_Mitosis	3.694E-02

HB2 exon7 siRNA

Cell cycle_G1-S Growth factor regulation	4.591E-07
Cardiac development_FGF_ErbB signaling	3.683E-05
Cardiac development_Role of NADPH oxidase and ROS	3.951E-05
Signal transduction_ERBB-family signaling	1.315E-04
Cell cycle_G2-M	1.539E-04
Cell adhesion_Amyloid proteins	1.923E-04
Inflammation_IL-2 signaling	2.201E-04
Cell cycle_G1-S Interleukin regulation	2.339E-04
Inflammation_MIF signaling	7.018E-04
Cell cycle_Mitosis	7.405E-04

SUM159 exon1 siRNA

	p value
Cytoskeleton_Actin filaments	2.179E-05
Cell cycle_Mitosis	9.478E-05
Cell adhesion_Integrin-mediated cell-matrix adhesion	7.909E-04
Cytoskeleton_Regulation of cytoskeleton rearrangement	1.166E-03
Cardiac development_Role of NADPH oxidase and ROS	1.595E-03
Signal Transduction_TGF-beta, GDF and Activin signaling	5.367E-03
Cell cycle_G1-S Growth factor regulation	5.667E-03
Apoptosis_Apoptotic nucleus	6.899E-03
Development_EMT_Regulation of epithelial-to-mesenchymal transition	8.326E-03
Cell cycle_G1-S	8.358E-03

SUM159 exon7 siRNA

Cell cycle_G1-S Growth factor regulation	4.637E-08
Cell cycle_G1-S Interleukin regulation	4.375E-06
Cell adhesion_Cadherins	4.066E-05
Apoptosis_Apoptotic mitochondria	4.515E-05
Cardiac development_Role of NADPH oxidase and ROS	1.623E-04
Cell cycle_G2-M	1.749E-04
Proliferation_Lymphocyte proliferation	2.035E-04
Signal transduction_ERBB-family signaling	2.310E-04
Cell adhesion_Amyloid proteins	3.615E-04
Cell adhesion_Glycoconjugates	8.858E-04

c

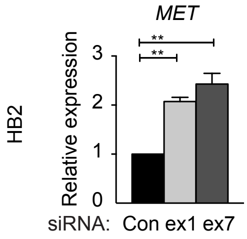

d

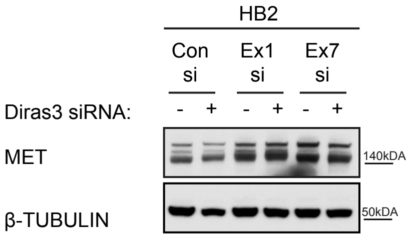

Supplementary Figure 10. Gene expression analysis reveals cellular pathways regulated by *GNG12-AS1*

a) Transcriptional profiling of HB2 (left panel) and SUM159 cells (right panel) with *GNG12-AS1* siRNA targeting exon 1 and exon 7 versus control siRNA with six biological replicates per group. Heatmaps depict expression data for genes found to be differentially expressed at FDR<0.05.

b) Metacore pathway enrichment analysis of differentially expressed genes. Top ten pathways for each comparison are displayed. Note that the cell cycle, cell adhesion and cytoskeleton are enriched with both *GNG12-AS1* siRNAs.

c) Upregulation of *MET* in HB2 cells after *GNG12-AS1* depletion with siRNAs targeting exon 1 and 7. Expression levels of *MET* were normalised to *GAPDH* and compared to control siRNA by qRT-PCR. The statistical significance of the individual bars is compared to their respective control bars. Error bars, s.e.m. (n = 3 biological replicates). p** < 0.01 by two-tailed Student's t-test.

d) Immunoblot of MET in HB2 cells after *GNG12-AS1* depletion with siRNAs targeting exons 1 and 7 or simultaneous depletion of *GNG12-AS1* and *DIRAS3*. Increase in MET levels are *DIRAS3* independent. β-tubulin is the same loading control as in Fig. 5 as MET immunoblot was done during the same experiment.

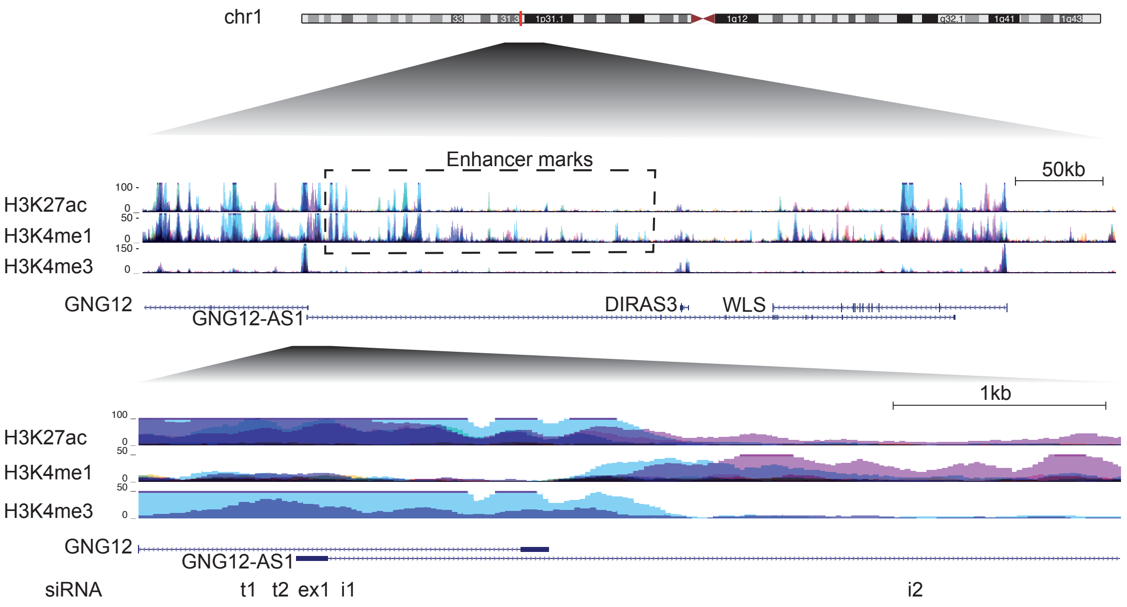

Supplementary Figure 11. The chromatin marks at the *GNG12-AS1* locus

The genome graphic data were taken from http://genome.ucsc.edu/ using the ENCODE data sets. Note the enrichment of enhancer modifications (H3K27ac, H3K4me1) downstream of *GNG12-AS1* TSS. siRNAs targeting *GNG12-AS1* promoter (t1, t2), exon 1 (ex1) or intronic region (i1, i2) are depicted.

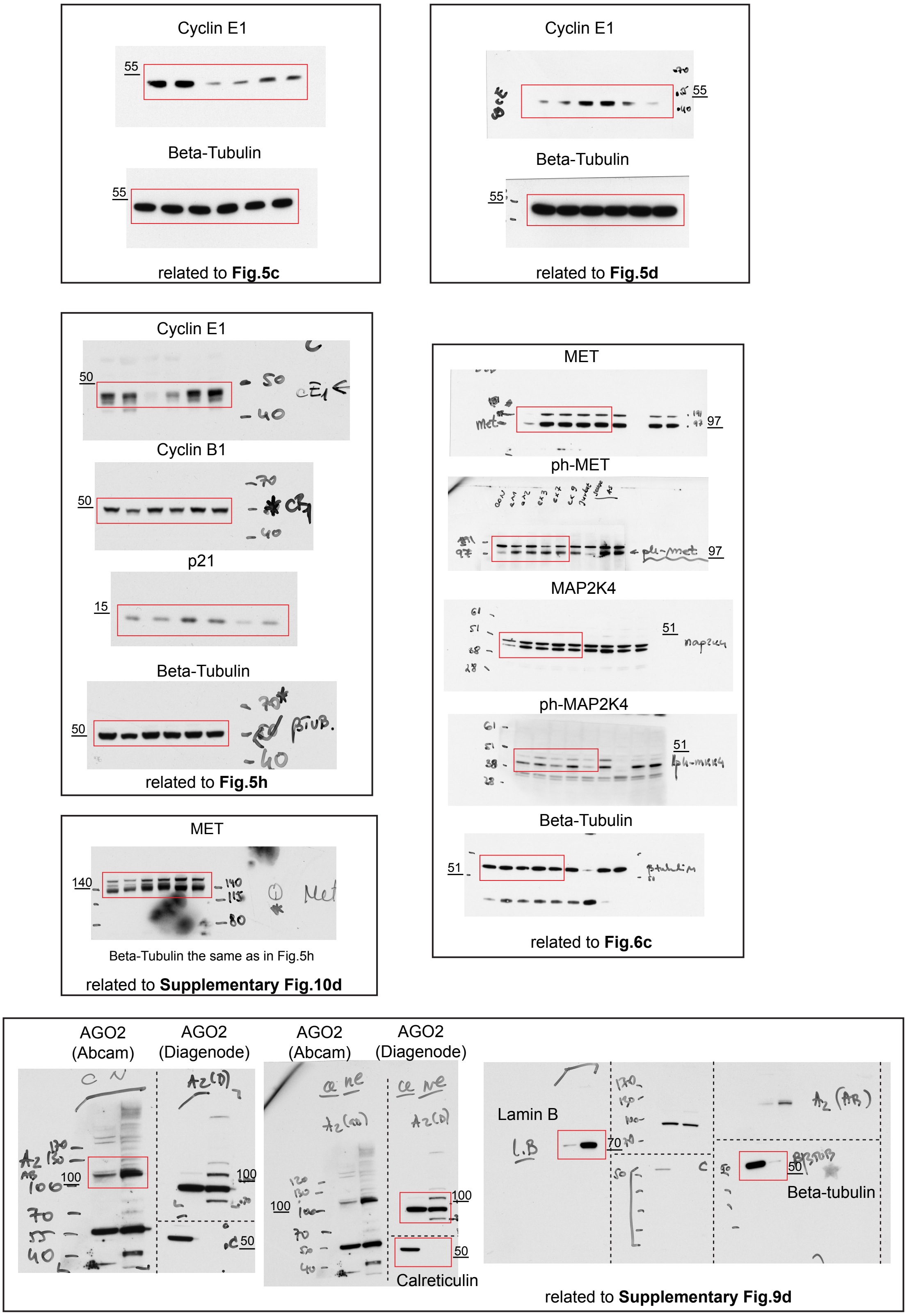

Supplementary Figure 12. Uncropped pictures of immunoblots

The red boxes regions are shown in the manuscript. Dotted lines indicate where a blot was cut to probe the same membrane with different antibodies.

Supplementary Tables

Supplementary Table 1. RNA FISH probes

GNG12-AS1 exonic probes(6e) Q570

GNG12-AS1_exonic_1	ggtcatctggtggtcatcct
GNG12-AS1_exonic_2	cccaactgggactgagtgga
GNG12-AS1_exonic_3	gccgcagtggagtgaaactt
GNG12-AS1_exonic_4	taaggaagccgtgattcttg
GNG12-AS1_exonic_5	attcccatgtgttgtggcag
GNG12-AS1_exonic_6	catcttaaattgtactccca
GNG12-AS1_exonic_7	gctctgtgttctcatccata
GNG12-AS1_exonic_8	gaagatagagttgatatggt
GNG12-AS1_exonic_9	tgagagaaatgtaaacaaac
GNG12-AS1_exonic_10	ttctgggccacaaaacatac
GNG12-AS1_exonic_11	tggaatgtttaccaggatca
GNG12-AS1_exonic_12	acaacagcagaatacacatt
GNG12-AS1_exonic_13	aaccatggattggaaatatt
GNG12-AS1_exonic_14	gatgacaaacagggaccaca
GNG12-AS1_exonic_15	gaagctgatggccgtattga
GNG12-AS1_exonic_16	ccacagtgcagaggaaaggt
GNG12-AS1_exonic_17	cagaggaaacacgagagctg
GNG12-AS1_exonic_18	gaagataagcgtccactgca
GNG12-AS1_exonic_19	gacaaagaaatgtgccatcg
GNG12-AS1_exonic_20	ttcctgtactagggcttgat
GNG12-AS1_exonic_21	ctggcacagattagtgtgaa
GNG12-AS1_exonic_22	gatgttcagatggcaggact
GNG12-AS1_exonic_23	taatctatggtgcatcaggc
GNG12-AS1_exonic_24	ccgagtgagtggtagacact
GNG12-AS1_exonic_25	gagtcttcatcttgtcttac
GNG12-AS1_exonic_26	gacactgaagttctgaagtc
GNG12-AS1_exonic_27	ggcgaagcatttaaaatcct
GNG12-AS1_exonic_28	gctccacaacaattgaggtt
GNG12-AS1_exonic_29	tcttcaaagctgttgcatct
GNG12-AS1_exonic_30	tggcgcttgtttgtcacgta
GNG12-AS1_exonic_31	caggcaagtaatgtaagcca
GNG12-AS1_exonic_32	ctccattccatgggatccaa
GNG12-AS1_exonic_33	ctagcaccaatcttgagcct
GNG12-AS1_exonic_34	acatccaatgattggcagat
GNG12-AS1_exonic_35	cactggattgctacaccaat
GNG12-AS1_exonic_36	ctttgctgcaggacaccaag
GNG12-AS1_exonic_37	cagtcttttcgatttaggct
GNG12-AS1_exonic_38	aattgcaggcatttttcagg
GNG12-AS1_exonic_39	gagcaatagcgtcaacagat
GNG12-AS1_exonic_40	agatgactggccagccgagc
GNG12-AS1_exonic_41	gcccaaagtcaaaacttagc
GNG12-AS1_exonic_42	attccacagtcttttggctg
GNG12-AS1_exonic_43	aattcactttccacaggtgg

GNG12-AS1_exonic_44	agcccaaggtagtaaattaa
GNG12-AS1_exonic_45	catgctgggtttctgacatc
GNG12-AS1_exonic_46	gctctgagagatttcccaca
GNG12-AS1_exonic_47	ctttgccagagccactttgg
GNG12-AS1_exonic_48	aggtgtcccctttttgtta

Intronic probes (Full intron) of GNG12-AS1 Q670

Full INTRON GNg12-AS1 _1	gtgtcctgtaaacttggctt
Full INTRON GNg12-AS1 _2	acagaaccaaactttcgtgt
Full INTRON GNg12-AS1 _3	ccagggccctttaaatgtta
Full INTRON GNg12-AS1 _4	ggttttctgaacatccagct
Full INTRON GNg12-AS1 _5	ccacactggttctaagtgac
Full INTRON GNg12-AS1 _6	agtccctagttcccagattc
Full INTRON GNg12-AS1 _7	ggcatgcattcatacaccta
Full INTRON GNg12-AS1 _8	caatatgtggtgttccagct
Full INTRON GNg12-AS1 _9	gaaaggacactggatcaagg
Full INTRON GNg12-AS1 _10	gatgcttgcttttcaaaccc
Full INTRON GNg12-AS1 _11	cgagagactgactgatttgc
Full INTRON GNg12-AS1 _12	ccatgtgcaataggaccaat
Full INTRON GNg12-AS1 _13	agcagaaaaaccagtttgga
Full INTRON GNg12-AS1 _14	tttcaacaggttacttgggg
Full INTRON GNg12-AS1 _15	cacccatgtaagtgtggaac
Full INTRON GNg12-AS1 _16	agccagagagctcatattga
Full INTRON GNg12-AS1 _17	aatttgccatgctgaaaacg
Full INTRON GNg12-AS1 _18	ctcttctgccaggacaattt
Full INTRON GNg12-AS1 _19	cctctgtatatctggaggct
Full INTRON GNg12-AS1 _20	ccttgctcatgtgagttctg
Full INTRON GNg12-AS1 _21	ctgagttctggaaatgagca
Full INTRON GNg12-AS1 _22	ctgacaaagagcgtgtttct
Full INTRON GNg12-AS1 _23	ggagtcaaaacccaatagca
Full INTRON GNg12-AS1 _24	tctgggtagtctcaagacag
Full INTRON GNg12-AS1 _25	tgcagactgtatttgcaagt
Full INTRON GNg12-AS1 _26	atgcgtgttttcctccatag
Full INTRON GNg12-AS1 _27	ttcattaggcagaactctgc
Full INTRON GNg12-AS1 _28	cggctacctaggaatactct
Full INTRON GNg12-AS1 _29	gtcctcagttggtgaaactc
Full INTRON GNg12-AS1 _30	aacctgaaacttgcttaggg
Full INTRON GNg12-AS1 _31	gtgtcccaggaaggaaaatt
Full INTRON GNg12-AS1 _32	acaagccgcttaagttactg
Full INTRON GNg12-AS1 _33	agttactaactgccagggta
Full INTRON GNg12-AS1 _34	cttctcttgcttgtctcgtg
Full INTRON GNg12-AS1 _35	gagcaaagctgatatcccaa
Full INTRON GNg12-AS1 _36	tattcccaggtgacctactc
Full INTRON GNg12-AS1 _37	gaaatatggcagtctccgac
Full INTRON GNg12-AS1 _38	atctgtagccacagttttgg

Full INTRON GNg12-AS1 _39	tcaactaacttgcatgggtg
Full INTRON GNg12-AS1 _40	tagagctagagggcctattg
Full INTRON GNg12-AS1 _41	catctgagggcaaactgtag
Full INTRON GNg12-AS1 _42	cctccaggtctacgtctatt
Full INTRON GNg12-AS1 _43	tttgatgctggatactgctg
Full INTRON GNg12-AS1 _44	cattgctcagactgccatta
Full INTRON GNg12-AS1 _45	aaggccaatatccagctttt
Full INTRON GNg12-AS1 _46	atcacaggctgcttttcttt
Full INTRON GNg12-AS1 _47	ttagcttacaggtccacaga
Full INTRON GNg12-AS1 _48	ctctgcaagtagatccatgc

Supplementary Table 2. List of primer sequences

Expression primers	Forward primer (5'to3')	Reverse primer (5'to3')
GAPDH	CAACAGCCTCAAGATCATCA G	ATGGACTGTGGTCATGAGTC
β-actin	GTTACACCCTTTCTTGACAAA	GTCACCTTCACCGTTCCAGTT
RPS18	ATCCCTGAAAAGTTCCAGCA	CCCTCTTGGTGAGGTCAATG
MALAT1	GACGGAGGTTGAGATGAAGC	ATTCGGGGCTCTGTAGTCCT
DIRAS3	TCTCCTCTTCAAATGCCAATG	GCGTGTAGAAAACGTGGACT C
GNG12	ACTCCTGGCATGTTCCTCAC	CAATATAGCCCAGGCAAGGA
WLS	CTTTCATGGGCCATTTCAGT	CCTTGGTTCCAATTCATGCT
GNG12-AS1 Exon 1-2	CAGTCCCAGTTGGGCAAAGT	GGCTCTGTGTTCTCATCCATA TCT
GNG12-AS1 Exon 1-3	CAGTCCCAGTTGGGCAAAGT	TACCAGGATCACATTCTGGGT CAC
GNG12-AS1 Exon 1-5	TCCCAGTTGGGCAAAGTTTC	CCTCTGTATCCGCAGGTTCC
GNG12-AS1 Exon 1-7	CAGTCCCAGTTGGGCAAAGT	TGGATGACAAACAGGGACCA
GNG12-AS1 Exon 5-7	CGGAACCTGCGGATACAGAG	GCTGATGGCCGTATTGACTG
GNG12-AS1 Exon 3-5	TGTGACCCAGAATGTGATCC	AGTCCTCTGTATCCGCAGGTT
GNG12-AS1 Exon 7-8	CCGATGGCACATTTCTTTGT	TGATGTTCAGATGGCAGGAC
GNG12-AS1 Exon 7-9 (for overexpression)	CCGATGGCACATTTCTTTGT	CATCTTTGGCTCCACAACAAT
GNG12-AS1 exon 7 (for intronic siRNA)	GTCAATACGGCCATCAGCTT	GGAAGATAAGCGTCCACTGC
C-MYC	GCCACGTCTCCACACATCAG	TCTTGGCAGCAGGATAGTCC T
Cyclin E1	TACACCAGCCACCTCCAGAC AC	CCTCCACAGCTTCAAGCTTTT G
Cyclin B1	TTTGCACTTCCTTCGGAGAGC	AAGGAGGAAAGTGCACCATG TC
U1 (RIP)	ATACTTACCTGGCAGGGGAG	CAGGGGGAAAGCGCGAACG CA
p21	CAT# QT00062090 (Qiagen)	
AGO1	CAT# QT00006370 (Qiagen)	
AGO2	CAT# QT00058408 (Qiagen)	
MET	CAT# QT00023408 (Qiagen)	
MAP2K4	CAT# QT00082530 (Qiagen)	

Primers used for Nuclear Run ON	Forward primer (5'to3')	Reverse primer (5'to3')
β-actin	AGGTCATCACCATTGGCAAT GAG	CTTTGCGGATGTCCACGTCA
1.6kb downstream from GNG12-AS1 TSS	GAATCAGACAAGGGGCTTGC	TCCGCTGAGTGGTTTTCTGA

(b)		
DIRAS3 TSS (d)	ACGAACCAAGCAGCCTAGAA	ATGCCTGTTACCCACACTCC
**Primers used for ChIP analysis**	**Forward primer (5'to3')**	**Reverse primer (5'to3')**
Gene dessert (negative control for Pol II and histone modifcations)	TGGTGGTCTGCCTTCTGCCA GT	TCACGTGGGAGGAAGAAGTA GGGC
GAPDH TSS (negative control for Ago2 ChIP)	CAGCCGCCTGGTTCAACTG	CCCAACTTTCCCGCCTCTC
GNG12-AS1 TSS (a)	GCTGGGAGCAGCCATTAAAC	CCGGTCATCTGGTGGTCAT
1.6kb downstream from GNG12-AS1 TSS (b)	GAATCAGACAAGGGGCTTGC	TCCGCTGAGTGGTTTTCTGA
DIRAS3 exon2 (c)	TCTCCTCTTCAAATGCCAATG	GCGTGTAGAAAACGTGGACT C
DIRAS3 TSS (d)	ACGAACCAAGCAGCCTAGAA	ATGCCTGTTACCCACACTCC
GNG12-AS1 ex7 rs11209218 (e)	GTCAATACGGCCATCAGCTT	GGAAGATAAGCGTCCACTGC
GNG12-AS1 ex8b (f)	AGTTCTCTGCTGCCATGTCTT T	GACACTGAAGTTCTGAAGTCT GGA
GNG12-AS1 ex9 rs7529246 (g)	TGTTTACATTTTAGGCAGATG AGG	CCAAGCCAGGCAAGTAATGT
**Primers used for Medip analysis**	**Forward primer (5'to3')**	**Reverse primer (5'to3')**
GNG12-AS1 TSS	GCTGGGAGCAGCCATTAAAC	CCGGTCATCTGGTGGTCAT
DIRAS3 (DMR1)	CCGATTGTGTCGTTCCTTTT	CTCACAGGCAAGGGAGAAAG
GNG12 TSS	GAACAGCTCCTGCACATTCA	CATAAGTTCCCCATCGTGCT
GNG12-AS1 ex7 rs11209218 (e)	GTCAATACGGCCATCAGCTT	GGAAGATAAGCGTCCACTGC
GNG12-AS1 ex9 rs7529246 (g)	TGTTTACATTTTAGGCAGATG AGG	CCAAGCCAGGCAAGTAATGT
**α** -satelite	CGATCCTTTACACAGAGCAG AC	AAGATATTTCCTATTCTACCA TTGACC

Supplementary Table 3. Primer efficiency of housekeeping genes in different cell lines

GAPDH	Slope	Y intercept	PCR efficiency	r^2
HB2	-3.448	22.003	95.00%	0.9999
MCF10A	-3.2568	19.763	102.79%	0.9992
HS27	-3.088	19.194	110.78%	1
SUM159	-3.1105	20.665	109.65%	0.9982
MCF7	-3.226	21.080	104.17%	0.9969

RPS18	Slope	Y intercept	PCR efficiency	r^2
HB2	-3.167	19.767	106.91%	0.998
MCF10A	-3.1998	19.352	105.36%	0.9945
HS27	-3.170	19.823	106.77%	0.9986
SUM159	-3.368	20.132	98.10%	0.9972
MCF7	-3.365	20.312	98.24%	0.9987

β-actin	Slope	Y intercept	PCR efficiency	r^2
HB2	-3.413	18.690	96.34%	0.9998
MCF10A	-3.5624	18.162	90.86%	0.9998
HS27	-3.330	17.503	99.68%	0.9994
SUM159	-3.547	19.700	91.38%	0.999
MCF7	-3.569	18.980	90.62%	0.9993

Supplementary Table 4. siRNA sequences

siRNA	sequence (Sense)	sequence (Antisense)	ID
GNG12-AS1 exon 1 siRNA #1 (Life Technologies, Silencer select) #S59962	CGGCGACGUGAACAAGAAU	AUUCUUGUUCACGUCGCCG	4392421
GNG12-AS1 exon 1 siRNA #2 (Life Technologies, Silencer select) #S59963	AAGAAUCACGGCUUCCUUA	UAAGGAAGCCGUGAUUCUU	4392421
GNG12-AS1 exon 7 siRNA#1 (Life Technologies, Silencer select) #S444122	GUGCUGCAGUGGACGCUUA	UAAGCGUCCACUGCAGCAC	4399666
GNG12-AS1 exon 7 siRNA#2 (Life Technologies, Silencer select) #S444121	CGGCGACGUGAACAAGAAU	AUUCUUGUUCACGUCGCCG	4392421
GNG12-AS1 exon 2 siRNA (Life Technologies, Silencer select) #S444299	GGAGUACAAUUUAAGAUGA	UCAUCUUAAAUUGUACUCC	4399666
GNG12-AS1 exon 3 siRNA (Life Technologies, Silencer select) #S444300	CAUUCCAUUUGAGAAGAAU	AUUCUUCUCAAAUGGAAUG	4399666
GNG12-AS1 exon 5-1 siRNA (Life Technologies, Silencer select) #S502698	GGACUGACUAUAAUAUCAA	UUGAUAUUAUAGUCAGUCC	4390827
GNG12-AS1 exon 5-2 siRNA (Life Technologies, Silencer select) #S502699	CGGAACCUGCGGAUACAGA	UCUGUAUCCGCAGGUUCCG	4390827
GNG12-AS1 exon 1 siRNA scrambled (Life Technologies, Silencer select) #S501057	AAUCGCAGCCGUAAUAGC	UGCUAUUACGGCUGCGAUU	4390827
GNG12-AS1 exon 7 siRNA scrambled (Life Technologies, Silencer select) #S501058	CGUUAAGCUACGCGUUGCU	AGCAACGCGUAGCUUAACG	4390827
GNG12-AS1 exon 1_C911 siRNA (Life Technologies, Silencer select) #S501055	CGGCGACGACUACAAGAAU	AUUCUUGUAGUCGUCGCCG	4390827
GNG12-AS1 exon 7_C911 siRNA (Life Technologies, Silencer select) #S501056	GUGCUGCACACGACGCUUA	UAAGCGUCGUGUGCAGCAC	4390827
Intronic siRNA against GNG12-AS1 (Life Technologies, Silencer select) #S502704 (195 bp downstream of TSS)	GGUGCUAAGUGACAGCGUU	AACGCUGUCACUUAGCACC	4390827
Intronic siRNA against GNG12-AS1 (Life Technologies) #S454585 SIRNA (2933bp downstream of TSS)	GCAACAGAUUGAAAUAA	UUUAUUUUCAAUCUGUUGC	4390827
GNG12-AS1 siRNA t1 (Life Technologies, Silencer select) #S502685 (129bp upstream of	ACUCCACACGGUCUAAUAA	UUAUUAGACCGUGUGGAGU	4390827

TSS)			
GNG12-AS1 siRNA t2 (Life Technologies, Silencer select) #S502686(33bp upstream of TSS)	GGUGUUCAGAGAUUUCCUU	AAGGAAAUCUCUGAACACC	4390827
Ago1 siRNA (Dharmacon)			M-004638-00
Ago2 siRNA (Dharmacon)			M-004639-00
DIRAS3 siRNA (Dharmacon)			M-008660-01
siGENOME Non-Targeting siRNA Pool #2 (Dharmacon)			001206-14-20
Negative control siRNA #1 (Life Technologies, Silencer select)			4390084

Supplementary Table 5. Primer sequences for pyrosequencing

Pyrosequencing assays for SNP analysis.

**Assay**	**SNP**	**Primer name**	**Sequence (5' to 3')**
DIRAS3	rs11801053	[Btn]DIRAS3_f	AATTCTCTGGCCTGGGAAAAA
		DIRAS3_r	TTTTGTGCTGTTGTTTGGACTGTA
		DIRAS3_S_r	TTGTTTGGACTGTAACATC

Pyrosequencing assays for analysis of DMR1, DMR2 and DMR3 methylation levels.

**Assay**	**Primer name**	**Sequence 5' to 3'**
DMR1	DMR1_f [btn]	ATTGAAGATATGTAGATGGTAAATAATTAT
	DMR1_r	AAAAAACAATACCAATAACCCACAC
	DMR1_S_	CCACACACTCCCCAA
DMR2	DMR2_f [btn]	TGGATATTTTAAGAGTTTATTTTTT
	DMR2_r	AACCAACCCCTCACAACTAATT
	DMR2_S_	CCTATTACCCACACTCC
DMR3	DMR3_f [btn]	TTTTAAGGAATAGAAGTTGTTGAAG
	DMR3_r	CCCAACAACTAACAATAAATATTTT
	DMR3_S_	CAATAAATATTTTCAATAAT

Supplementary Table 6. List of antibodies

Antibodies	Catalog number
RNA polymerase II	Ab5408 Abcam
H3K9me3	Ab8898 Abcam
H3K4me3	Ab8580 Abcam
H3K27me3	Cell Signalingg 9733
H3K36me3	Ab9050 Abcam
Histone H3	Ab1791 Abcam
CYCLIN E1	Cell Signaling 4129 (dilution 1:1000 for Western)
CYCLIN B1	Cell Signaling 12231 (dilution 1:1000 for Western)
p21	Santa Cruz sc-6246 (dilution 1:1000 for Western)
β-tubulin	Sigma T0198 (dilution 1:2000 for Western)
5-mC antibody	MAb-006, Diagenode
Control rabbit IgG	Cell Signaling 2729
Control mouse IgG	Cell Signaling 5415
AGO2	Diagenode 167-100 (ChIP)<br>(dilution 1:500 for Western)
AGO2	Abcam ab57113 (RIP)<br>(dilution 1:500 for Western)
Calreticulin	Cell Signaling 2891 (dilution 1:1000 for Western)
Lamin B1	Ab16048 Abcam (dilution 1:1000 for Western)
MET	Cell Signaling 8198 (dilution 1:1000 for Western)
phospho-MET (Tyr1234/1235)	Cell Signaling 3077 (dilution 1:1000 for Western)
MAP2K4	Cell Signaling 9152 (dilution 1:1000 for Western)
phospho-MAP2K4 (Ser257/Thr261)	Cell Signaling 9156 (dilution 1:1000 for Western)
