# Supplementary material for: Transcriptional silencing of long noncoding RNA GNG12-AS1 uncouples its transcriptional and product-related functions
Source: Nat Commun. 2016 Feb 2;7:10406. doi: 10.1038/ncomms10406 (PMC4740813; doi:10.1038/ncomms10406)
Supplement: Supplementary Data 1 — Sequences of the GNG12-AS1 splice variants and scrambled vector [file ncomms10406-s2.docx]

**Supplementary Data**

**Sequence of scrambled clone**

TGCCATTACAAATCCGATCCGGCAAAACTGTTGCTTGGGGGACGACTGGGCAACATCCGAGTGGAGCTACTCCTGGCCTCCCTGCTGGGACAAAAGGGTTCGTGGGTACACTTGTTTGTACTGCTTACCTTACAATTGTCTCTGGATTTGGAGAGAGATTGGGACTGGCCTCCACAATTGTACTGCAAGTACTGCACGTACAAGGACCATCGGAGACTCTATTGGGAATCGAGCTGCTTTGGGATTGTATCTCGTCAGGGTATCGGAGCACTCACTTGGAATTACGCCAAAAAATACGGCTTGCTTACTTGGTACTTGCGCCGTACTCTGCGCTGCATCCCTACTTACCACTGCTCATCCATTGCGTACTCTCTCGCGCACACTGTCAAGGATACTTATCCTCGCCCCGAAGGAAGTTCTTGCAAGGGAATCTGCACCGTTTACCATCAACGCGATCAAAGGCAAGCAGTCTCCTCGATACACTGTTTACACAATTGCTACCGGTCTCTGCGATACCTACCCGTCGTACAAACGCACACGTTCCGAACGTGCGTGGAGAATCGATTTTGGATTGTGCACATTTCTGCGACTGTACTACCATTCGTTTACTCCAAAGCCCCTGCAAAACCTTTCTATCTCGCTGCGGTGTCGAGCTATCCAATCTTACACTTTCTGCTTATCAACGTTACAGCACGTACTACTCGAGATACATCTTACTGCATTGCTGGGCTACCCACACACCAGGTGCTCAAACGACACAATACCGTGGCGGCGATTGCATCAACACTGGTGTCTCCGGGAGCCGGACGCGACAGGTGGTGTTCCCATTATTGCAGTGGAGAATACTATATTGGGTTGGATTCCCCTTGGCCTACGTGCGTGGCCTCCATCTATTCCCCCCTCTGCACGGACACTTGTTCCTACAGTGTCTTTATACGGGTCTCACTCCATTACAACACAAAGTGTTTACGTCGCGACTACTCCTTGCATACAAGTGCAATACTCCCCGTGCGTCATTATACGTGCGAGGGACACCAGCCAGGTTACAACAGCGCCGACCTGTGGACGTACCCGAGCAATCGCGTACCATCTCATCCCTTCTTTCTGCTACCGTGGGAGTGGCCGGTACGTGCCACTGCAAAAAGAACACATCTCTACTATATCATCCTGGAACGCCCTATACGTATCAGGGAGGGTGCCACAGGGCCAGAGACCCTGCCCCCATACACTTCGTCGTGGGGTTCCGTCTTGGATACATCGAAGAACGTCAAGCGATACGTACCCTTCAACTATCCTACGGTTCATTTCAATTTATCCTACACGCAGGTGCAGCCCTTTTTGCTTACAGTGGAGGCAATACTTCAGTCTCCAACGCGGCAAAACTTTTCGGGTTGCGCTGGTTGGTATCAGATCGCCGTGGCCGCGACCGTACTCAAGAGTTTGCGTTGGCACAGGTGCGGAGTACCAACAATTCAATTTTTGTACGTCTCAAGCGTTCAGGAGTACCTCGAATTACTCCTATCGATCACACATACTCTTACAGCTACGTGCAGCCTGGGACACTGGAAATCATTGCCCTTGGGGTACTCATCTTCTCGCCTGTACTACAATCGCATCATACTCTGGCCACCATTACACTTACTCACAACCTCGGAGGAATCGTTACGAACGAAACCCCACCAGGTCCGTTGGTGGCAGTGGGAATCCTCCGTGGGTTTCCCGCAAATCACTACTCAATTGGGGATCTACCTTTTTACGGATCTACTACTACACGAACACCTACAGCAATTGTTGCGCCTGTATACAGCCTTATTATTACCTTGGCTGTCGAAGCAAGTTTCTTCAGCTCGACGCCTCCCACGCGACGGGAAGGCACATTGTGCTTCTGCCTACACACGTGGCCTGTATCTGCACTCACTGCAACGATTGGTCCCATTCCAGCCCTGCTCCTGTAC

**Sequence of *GNG12-AS1* variant 1 (clone 1-2-3-5-5b-7-8-8b-9)**

TTGTATACAACCACGCCCGGCAGGATGACCACCAGATGACCGGCCGCAGCAATGCCTCCACTCAGTCCCAGTTGGGCAAAGTTTCACTCCACTGCGGCGACGTGAACAAGAATCACGGCTTCCTTATCGTACCTGCCACAACACATGGGAATTATGGGAGTACAATTTAAGATGAGATATGGATGAGAACACAGAGCCAAACCATATCAACTCTATCTTCATGTTTGTTTACATTTCTCTCAACTGTATGTTTTGTGGCCCAGAATGTGATCCTGGTAAACATTCCATTTGAGAAGAATGTGTATTCTGCTGTTGTTGGAATATTTCCAATCCATGGTTGGGTGCGGAACCTGCGGATACAGAGGACTGACTATAATATCAAGGAGGCTCGTGTGGTCCCTGTTTGTCATCCAGTCAATACGGCCATCAGCTTCTGGCCCCAGACACCTTTCCTCTGCACTGTGGCACAGAGGAGCAGACTGCTTTGGAGGGCAGCTTCCTGGGAGCTCCAGGAACCCTGGCCAGGCGGCCACTGCCACTGACTTCCTCGGCTCACTGAGCCCGTGCCTGCAGCTCTCGTGTTTCCTCTGTGCTGCAGTGGACGCTTATCTTCCCCCGATGGCACATTTCTTTGTCACAGACAGGACCCTCACTGGCAGTCGGAGGACATCAAGCCCTAGTACAGGAAGGGACATTTTCACACTAATCTGTGCCAGCTGGAGCAGTCCTGCCATCTGAACATCATATTTTGGAACCCTGAGAAGCTAGGATGCAGTTAACAGCTAGATGTCAATTCCCTGCAGGCAGAGACCAGTTCTCTGCTGCCATGTCTTTAAGGCCTGATGCACCATAGATTAATTCAAATCCAGTGTCTACCACTCACTCGGCTTAATGTAAGACAAGATGAAGACTCCAGACTTCAGAACTTCAGTGTCCGCAGATGAGGATTTTAAATGCTTCGCCTACAACCTCAATTGTTGTGGAGCCAAAGATGCAACAGCTTTGAAGAATAGAAGAATTACTCTATATTTGTCAGGATCTACGTGACAAACAAGCGCCACTTGGCTTACATTACTTGCCTGGCTTGGATCCCATGGAATGGAGGAGCATTGGAACCAAGAGGAAAAAAATTAAAGTGCTTTGGGACAGCGAGCCAAACTCTTACATAGTATTTGAAATGGTGTGGCAAACATGAAATTATTTTAGGCTCAAGATTGGTGCTAGTTTTTTACCCTCTTTTTGAAAAAATAGTAAAATGCACATAAAGGGCCCTGCCAGAGGTTTAGCTCATAAAATCTGCCAATCATTGGATGTGCTAATTGGTGTAGCAATCCAGTGGTGGCCTGGATTAGGACTCAAAAACTGACCCTTACCACCCTTGGTGTCCTGCAGCAAAGACAGCCTAAATCGAAAAGACTGATGGAAGTATATCAAATTACGAATTTCTCTATCCCTGAAAAATGCCTGCAATTTCTGTTTCTCCATCACTCCAGAAATACTCAAAACAGCAAAAATTAAATACATATGAAGTGAACACAAAAGACCTCCACAGAAAAATCAGTGGCTCATCTGTTGACGCTATTGCTCATTGCTCGGCTGGCCAGTCATCTGGGCTAAGTTTTGACTTTGGGCCAGTGCTTCAAAATCAGCAACCTTTCAGCCAAAAGACTGTGGAATGCAGCCAAGACCACCTGTGGAAAGTGAATTATAGCATCTTTAATTTACTACCTTGGGCTATGATGTCAGAAACCCAGCATGGAGGGCGCACCAGTTCTTGTGTGGGAAATCTCTCAGAGCCCCCATGGAGGCCCCCAAAGTGGCTCTGGCAAAGCTGTAGGGGTGGAGGTAACAAAAAAGGGGACACCTGGCTCTCCTTCTTAAATCAGCTCACCATGGGCACACATTTATATTGGAATTTTAGGGTCAGAAAATACCAAAATTAAATCTTCTAGAAG

**Sequence of *GNG12-AS1* variant 2 (clone 1-5-5b-7-8-8b-9)**

TTGTATACAACCACGCCCGGCAGGATGACCACCAGATGACCGGCCGCAGCAATGCCTCCACTCAGTCCCAGTTGGGCAAAGTTTCACTCCACTGCGGCGACGTGAACAAGAATCACGGCTTCCTTATCAATATTTCCAATCCATGGTTGGGTGCGGAACCTGCGGATACAGAGGACTGACTATAATATCAAGGAGGCTCGTGTGGTCCCTGTTTGTCATCCAGTCAATACGGCCATCAGCTTCTGGCCCCAGACACCTTTCCTCTGCACTGTGGCACAGAGGAGCAGACTGCTTTGGAGGGCAGCTTCCTGGGAGCTCCAGGAACCCTGGCCAGGCGGCCACTGCCACTGACTTCCTCGGCTCACTGAGCCCGTGCCTGCAGCTCTCGTGTTTCCTCTGTGCTGCAGTGGACGCTTATCTTCCCCCGATGGCACATTTCTTTGTCACAGACAGGACCCTCACTGGCAGTCGGAGGACATCAAGCCCTAGTACAGGAAGGGACATTTTCACACTAATCTGTGCCAGCTGGAGCAGTCCTGCCATCTGAACATCATATTTTGGAACCCTGAGAAGCTAGGATGCAGTTAACAGCTAGATGTCAATTCCCTGCAGGCAGAGACCAGTTCTCTGCTGCCATGTCTTTAAGGCCTGATGCACCATAGATTAATTCAAATCCAGTGTCTACCACTCACTCGGCTTAATGTAAGACAAGATGAAGACTCCAGACTTCAGAACTTCAGTGTCCGCAGATGAGGATTTTAAATGCTTCGCCTACAACCTCAATTGTTGTGGAGCCAAAGATGCAACAGCTTTGAAGAATAGAAGAATTACTCTATATTTGTCAGGATCTACGTGACAAACAAGCGCCACTTGGCTTACATTACTTGCCTGGCTTGGATCCCATGGAATGGAGGAGCATTGGAACCAAGAGGAAAAAAATTAAAGTGCTTTGGGACAGCGAGCCAAACTCTTACATAGTATTTGAAATGGTGTGGCAAACATGAAATTATTTTAGGCTCAAGATTGGTGCTAGTTTTTTACCCTCTTTTTGAAAAAATAGTAAAATGCACATAAAGGGCCCTGCCAGAGGTTTAGCTCATAAAATCTGCCAATCATTGGATGTGCTAATTGGTGTAGCAATCCAGTGGTGGCCTGGATTAGGACTCAAAAACTGACCCTTACCACCCTTGGTGTCCTGCAGCAAAGACAGCCTAAATCGAAAAGACTGATGGAAGTATATCAAATTACGAATTTCTCTATCCCTGAAAAATGCCTGCAATTTCTGTTTCTCCATCACTCCAGAAATACTCAAAACAGCAAAAATTAAATACATATGAAGTGAACACAAAAGACCTCCACAGAAAAATCAGTGGCTCATCTGTTGACGCTATTGCTCATTGCTCGGCTGGCCAGTCATCTGGGCTAAGTTTTGACTTTGGGCCAGTGCTTCAAAATCAGCAACCTTTCAGCCAAAAGACTGTGGAATGCAGCCAAGACCACCTGTGGAAAGTGAATTATAGCATCTTTAATTTACTACCTTGGGCTATGATGTCAGAAACCCAGCATGGAGGGCGCACCAGTTCTTGTGTGGGAAATCTCTCAGAGCCCCCATGGAGGCCCCCAAAGTGGCTCTGGCAAAGCTGTAGGGGTGGAGGTAACAAAAAAGGGGACACCTGGCTCTCCTTCTTAAATCAGCTCACCATGGGCACACATTTATATTGGAATTTTAGGGTCAGAAAATACCAAAATTAAATCTTCTAGAAG
